# Mucosal vaccination with attenuated *Mycobacterium tuberculosis* induces strong central memory responses and protects against tuberculosis

**DOI:** 10.1038/ncomms9533

**Published:** 2015-10-13

**Authors:** Deepak Kaushal, Taylor W. Foreman, Uma S. Gautam, Xavier Alvarez, Toidi Adekambi, Javier Rangel-Moreno, Nadia A. Golden, Ann-Marie F. Johnson, Bonnie L. Phillips, Muhammad H. Ahsan, Kasi E. Russell-Lodrigue, Lara A. Doyle, Chad J. Roy, Peter J. Didier, James L. Blanchard, Jyothi Rengarajan, Andrew A. Lackner, Shabaana A. Khader, Smriti Mehra

**Affiliations:** 1 Tulane National Primate Research Center, Covington, Louisiana 70433, USA; 2 Department of Microbiology and Immunology, Tulane Health Sciences Center, New Orleans, Louisiana 70112, USA; 3 Biomedical Sciences Graduate Program, Tulane Health Sciences Center, New Orleans, Louisiana 70112, USA; 4 Yerkes National Primate Research Center, Atlanta, Georgia 30329, USA; 5 Emory Vaccine Center, Atlanta, Georgia 30329, USA; 6 University of Rochester Medical Center, Rochester, New York 14642, USA; 7 Department of Pathology, Tulane Health Sciences Center, New Orleans, Louisiana 70112, USA; 8 Department of Molecular Microbiology, Washington University at St Louis, St Louis, Missouri 63110, USA; 9 Center for Biomedical Research Excellence, Louisiana State University School of Veterinary Medicine, Baton Rouge, Louisiana 70803, USA; 10 Department of Pathobiological Sciences, Louisiana State University School of Veterinary Medicine, Baton Rouge, Louisiana 70803, USA

## Abstract

Tuberculosis (TB) is a global pandaemic, partially due to the failure of vaccination approaches. Novel anti-TB vaccines are therefore urgently required. Here we show that aerosol immunization of macaques with the *Mtb* mutant in SigH (MtbΔ*sigH*) results in significant recruitment of inducible bronchus-associated lymphoid tissue (iBALT) as well as CD4^+^ and CD8^+^ T cells expressing activation and proliferation markers to the lungs. Further, the findings indicate that pulmonary vaccination with MtbΔ*sigH* elicited strong central memory CD4^+^ and CD8^+^ T-cell responses in the lung. Vaccination with MtbΔ*sigH* results in significant protection against a lethal TB challenge, as evidenced by an approximately three log reduction in bacterial burdens, significantly diminished clinical manifestations and granulomatous pathology and characterized by the presence of profound iBALT. This highly protective response is virtually absent in unvaccinated and BCG-vaccinated animals after challenge. These results suggest that future TB vaccine candidates can be developed on the basis of MtbΔ*sigH*.

## Abstract (web summary)

BCG, the only vaccine currently used against tuberculosis, confers only limited protection. Here the authors show that mucosal immunization of macaques with an attenuated strain of *Mycobacterium tuberculosis* confers a high level of protection from a lethal challenge with the bacterium.

## Graphical abstract

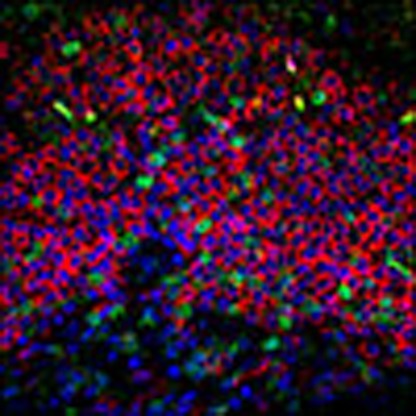

Despite widespread use of the bacille Calmette-Guérin (BCG) vaccine, *Mycobacterium tuberculosis* (*Mtb*) infection and the resulting incidence of tuberculosis (TB) remains a major global concern. The BCG vaccine, to a large extent and with some exceptions, mitigates only the most severe aspects of infection and exhibits a highly variable efficacy, especially in high-burden areas^1^. The majority of *Mtb*-infected individuals, including BCG-vaccinated ones, develop persistent but asymptomatic TB infection following *Mtb* exposure, rather than sterilizing immunity^2^. These individuals retain a finite risk of reactivation because of comorbidities such as HIV or diabetes. Developing new and efficacious TB vaccines is clearly the most effective intervention for containing the TB pandaemic^3^,^4^,^5^. To this end, a number of novel candidates are currently being evaluated either as potential replacements for BCG or to boost BCG-generated responses using a variety of approaches^6^. However, a frontline candidate that attempted to boost existing BCG responses failed to protect a target population against TB in a high-burden setting^7^. These results provide further impetus to the objective of replacing BCG with a new live attenuated vaccine.

The failure to generate an effective TB vaccine is attributed to a lack of specific immune correlates of protection from *Mtb* infection and TB disease. Of interest are adaptive responses, particularly pathogen-specific memory CD4^+^ and CD8^+^ T-cell-mediated responses essential for successful bacterial control during LTBI. BCG is generally considered to be inefficient in generating central memory CD4^+^ and CD8^+^ T-cell responses^8^, although long-term immunity following BCG administration is possible in some settings^9^. This contributes to the widely perceived inability of the vaccine to confer long-term protection against TB^10^. Thus, understanding related immune parameters and how perturbation by comorbidities leads to TB may be useful for effective TB vaccine design.

Aerosol delivery of BCG to the lung enhances protective efficacy^11^, including in macaques^12^. Further, aerosol TB vaccination might allow co-delivery of vaccine and adjuvants^13^. Aerosol–BCG resulted in significantly improved protection in guinea pigs against *Mtb* challenge compared with conventional vaccination^11^. Besides, an adenoviral vector expressing *Mtb* antigens elicited robust responses following aerosol delivery^14^.

Attenuated mycobacteria have evoked interest as potential BCG-replacement vaccines^15^. The *Mtb* antigen repertoire counterintuitively evokes strong immunity, suggesting that such responses work in the pathogen’s favour^16^. Immune-evasive pathways of *Mtb* are therefore the key to understand mechanisms of *Mtb* persistence^17^. SigH orchestrates a key stress-response pathway in *Mtb* that mitigates oxidative stress through induction of antioxidant production^18^. The *Mtb* mutant in *sigE*, which is part of the SigH regulon, elicited protection from *Mtb* infection^19^. Further, the *M. avium paratuberculosis sigH* mutant is being evaluated as a candidate vaccine for protecting cattle (US20140271719A1 (Pending US Patent, Adel Talaat)). Besides, aerosol immunization of macaques with the MtbΔ*sigH* mutant failed to cause disease^20^. This was in direct contrast to the phenotype of this mutant in C57Bl/6 mice, in which the *sigH*-null strain replicated to levels comparable to those of parental *Mtb*^18^. While the MtbΔ*sigH* mutant in H37Rv did not have a phenotype in monocytes^21^, the mutant in the CDC1551 strain exhibited growth restriction in bone marrow-derived macrophages (BMDMs)^22^. These results suggest that MtbΔ*sigH* fails to neutralize host-generated oxidants *in vivo* and is controlled in an elite manner by the primate innate immune system. Macaques accurately model several aspects of the human TB syndrome including partial protection from BCG vaccination^23^,^24^, different outcomes^25^ and the full spectrum of pathology^26^, and have the unique capacity to model *Mtb*/HIV co-infection^28^,^29^. A World Health Organization working group strongly recommended validation of candidate live TB vaccines in this model^30^.

In the current study, aerosol vaccination with MtbΔ*sigH* elicited strong CD4^+^ and CD8^+^ central memory T-cell responses as well as a robust T-helper 1 (Th1) response correlating with significantly greater protection from lethal *Mtb* challenge in rhesus macaques. Furthermore, protection from lethal TB in MtbΔ*sigH*-vaccinated animals was characterized by the presence of highly organized bronchus-associated lymphoid tissue (iBALT) associated with granulomas, following *Mtb* challenge.

## Results

### MtbΔ*sigH* infection and increased iBALT

**Figure 1:**
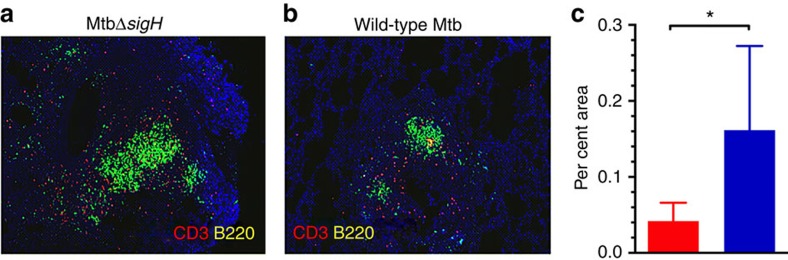
iBALT formation induced by MtbΔ*sigH* vaccination. Co-staining with CD3 and B220 revealed that lung granulomas following infection with MtbΔ*sigH* (**a**) exhibited a significantly increased iBALT response relative to infection with *Mtb* (**b**). White scale bar, 500 μm. Data from multiple lesions from six different animals were used in the analyses. (**c**) The percentage of area occupied by iBALT follicles relative to total lung area was analysed in animals challenged with *Mtb* (red) and MtbΔ*sigH* (dark blue). *P<0.05 (Student’s *t*-test). Data are means±s.d. Samples from four to five animals in each group were used for analysis.

We have previously established a correlation between the levels of ectopic lymphoid structures known as iBALT and the control of *Mtb* infection in a latent state. Experimentally infected macaques had significantly greater levels of iBALT in their lungs during LTBI, and this response was markedly reduced during TB disease^31^. iBALT was characterized by the presence of CD20^+^ B and CXCR5^+^ T cells. The expression of CXCR5 on T cells within iBALT follicles governed correct orientation and localization of T cells and macrophage activation^31^. Since aerosol infection of macaque lungs with MtbΔ*sigH* resulted in the virtually complete clearance of infection, we used samples from this previous study^20^ to assess whether the lungs of MtbΔ*sigH*-infected macaques harboured increased iBALT. Lung sections from six macaques, each exposed to high doses of *Mtb* CDC1551 strain or the isogenic MtbΔ*sigH* mutant, were co-stained for expression of CD3 and CD45R (B220)^31^. Immunofluorescence revealed that lung lesions from animals infected with the mutant contained significantly greater iBALT signal (Fig. 1a), relative to those infected with *Mtb* (Fig. 1b). The percentage of area occupied by iBALT was significantly greater in samples from MtbΔ*sigH*-infected than *Mtb*-infected animals (*P*<0.05; Fig. 1c). In addition, the total lung area involved in iBALT follicles (μm^2^; *P*<0.01) and the average size of these iBALT follicles (μm^2^; *P*<0.0001, Student’s *t*-test) was also significantly greater in sections derived from MtbΔ*sigH*-infected, relative to *Mtb*-infected animals (Supplementary Fig. 1a,b). These results further support our previous observations that protection from *Mtb* infection directly correlates with the presence of granuloma-associated iBALT and suggested that the lungs of MtbΔ*sigH*-infected macaques could exemplify an environment conducive to protection from TB.

### Analysis of aerosol-MtbΔ*sigH* as an anti-TB vaccine candidate

**Figure 2:**
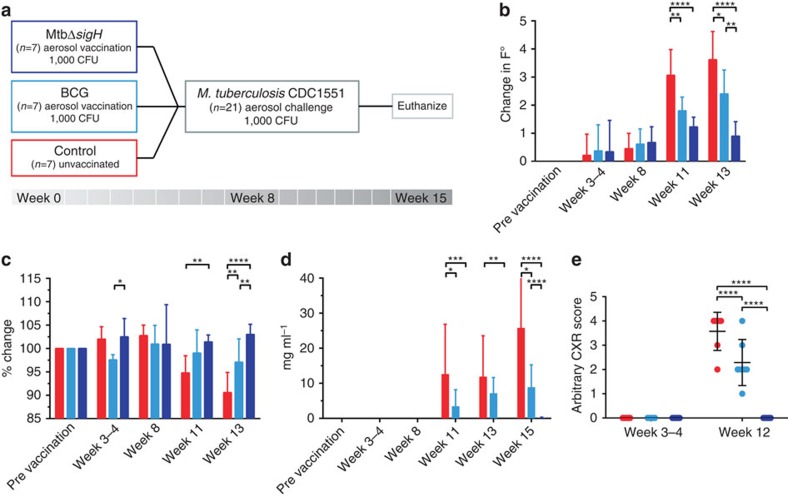
Study outline and clinical correlates of vaccination and infection. (**a**) Three groups of seven macaques were used: unvaccinated (red); vaccinated with BCG (light blue) and vaccinated with MtbΔ*sigH* (dark blue). (**b**) Changes (Δ°F) in body temperature; (**c**) changes in percentage of body weight; (**d**) changes in serum CRP (μg ml^−1^) levels; and (**e**) changes in relative thoracic radiograph (CXR) scores, over the course of the vaccination and infection phases. CXRs were scored in a blinded manner by categorizing between zero and four based on increasing involvement in the granulomatous pathology. *P<0.05, **P<0.01, ***P<0.001, ****P<0.0001 using two-way ANOVA with Tukey’s correction for multiple comparisons. Data are means±s.d. Samples from all seven animals in each group were used for analysis at each time point.

Encouraged by the magnitude of iBALT induction in macaques infected with MtbΔ*sigH*, we conceived a vaccine study to assess both the immunogenicity as well as the efficacy of this mutant as a potential vaccine against pulmonary TB. The design of the macaque study is outlined in Fig. 2a. Since enhanced iBALT responses were observed in the lungs of animals infected with the mutant strain via the aerosol route, we postulated that vaccination via the same route would have a greater chance of eliciting protection. Furthermore, BCG vaccination is more effective via the pulmonary route, indicating that local responses, elicited by matching the route of the vaccination to that of infection, may be critical to protect against TB^11^,^12^. Before and following single aerosol vaccination with MtbΔ*sigH* at a dose that elicited strong iBALT response^20^, both peripheral blood and lung compartments were repeatedly sampled to obtain cells for immune studies and transcriptomics. Eight weeks post vaccination, animals were challenged via aerosol, with a highly lethal dose of *Mtb*. Unvaccinated and BCG (aerosol)-vaccinated groups were included as appropriate controls (Fig. 2a). All animals that received aerosol–BCG or MtbΔ*sigH* vaccination became tuberculin skin test (TST) positive (Supplementary Table 1).

### Absence of disease on vaccination with MtbΔ*sigH* or BCG

Aerosolization of broth-cultured, log-phase BCG and MtbΔ*sigH* was performed as described in the Methods section^20^,^25^,^29^,^32^,^33^. Microbial efficiency of BCG and MtbΔ*sigH* was highly equivalent during aerosolization. Aerosol vaccination of macaques with MtbΔ*sigH* and BCG deposited ∼1,000 colony-forming unit (CFU) bacilli into the deep lung. Aerosol vaccination with either strain resulted in a positive TST (Supplementary Table 1) but did not induce dyspnoea, anorexia or significant changes in body temperatures relative to pre-infection values (Fig. 2b). The body weights of all vaccinated animals also remained relatively normal during the post-vaccination/pre-challenge phase with the exception of a slight decline (<3%) in the body weights of BCG-vaccinated animals 3–4 weeks post vaccination (Fig. 2c). None of the vaccinated animals exhibited an increase in serum C-reactive protein (CRP) levels relative to unvaccinated animals at any time post vaccination (Fig. 2d). Further, thoracic radiographs acquired post vaccination on all 14 vaccinated animals were normal (Fig. 2e).

### Differential persistence of MtbΔ*sigH* and BCG in BAL

**Figure 3:**
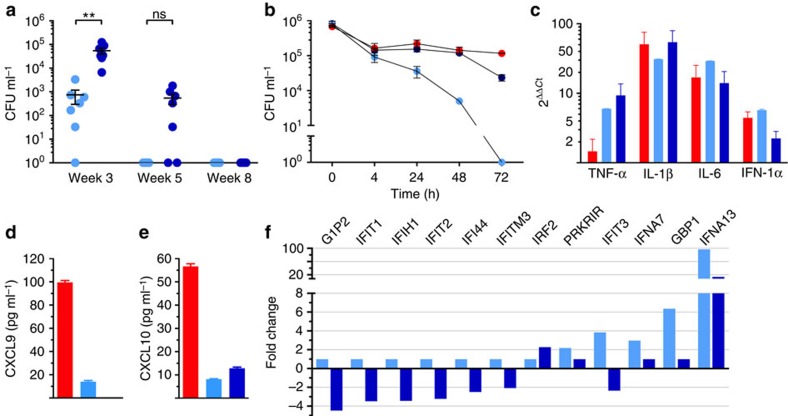
Comparative measures of bacterial burden in BAL following aerosol vaccination and restriction during intraphagosomal culturing *in vitro*. (**a**) BCG (light blue) and MtbΔ*sigH* (dark blue) CFU levels in total BAL samples at weeks 3, 5 and 8 after vaccination. (**b**) Rhesus macaque BMDM *in vitro* killing assay in CFU ml^−1^ with *Mtb* (red), BCG and MtbΔ*sigH*. **P<0.01 using two-way ANOVA with Tukey’s correction for multiple comparisons. Data are means±s.d. (**c**) Relative expression (2^ΔΔ*C*t^) of TNF-α, IL-1β, IL-6 and IFN-1α in BMDMs infected with *Mtb*, BCG and MtbΔ*sigH* using real-time RT–PCR. (**d**,**e**) Absolute expression of CXCL9 (**d**) and CXCL10 (**e**; pg ml^−1^) in supernatants derived from BMDMs infected with *Mtb*, BCG and MtbΔ*sigH* using cytokine analysis assay. (**f**) Microarray-derived fold changes of gene expression of 12 Type I interferon genes in the BAL of BCG- and MtbΔ*sigH*-vaccinated animals. For analysis involving BAL, samples from all seven animals in each group were used for analysis at each time point (**a**). For CFU analysis *in vitro*, the experiment was performed twice, with four biological replicates in each instance (**b**). Transcript and cytokine analyses were performed on biological replicates (**c**–**f**). ns, not significant.

The persistence of live mycobacteria was evaluated in bronchoalveolar lavage (BAL) from both MtbΔ*sigH*- and BCG-vaccinated macaques. Three weeks after vaccination, greater levels of MtbΔ*sigH* were recovered from BAL compared with BCG (Fig. 3a). At week 5 post vaccination, BCG could not be recovered from BAL, while detectable levels of MtbΔ*sigH* were still recovered (Fig. 3a), indicating that this strain might persist longer than BCG in human lungs. Eight weeks after vaccination, CFU for neither strain was recovered from the BAL of vaccinated macaques. Furthermore, macaque BMDMs were able to kill BCG at a faster rate than both *Mtb* and MtbΔ*sigH in vitro* (Fig. 3b). BMDMs infected with MtbΔ*sigH* expressed greater levels of tumour-necrosis factor-α (TNF-α) and interleukin (IL)-1β but not IL-6 transcripts relative to BCG (Fig. 3c). In addition, BMDMs infected with MtbΔ*sigH* also secreted significantly lower levels of inflammatory chemokines CXCL9 (Fig. 3d) and CXCL10 (Fig. 3e) in supernatants, relative to the BCG-vaccinated or the unvaccinated groups. Both the expression and anti-*Mtb* activity of TNF-α is strongly enhanced by IL-1β via direct augmentation of caspase-dependent apoptosis^34^. Infection of BMDMs with MtbΔ*sigH* results in both higher TNF-α expression and greater apoptosis relative to *Mtb*^22^. The activity of IL-1β is itself regulated by Type I interferons, whose expression is positively controlled by IRF1 and negatively by IRF2. Elicitation of Type I interferon response inhibits IL-1β and TNF-α activity, and promotes the progression of active TB, as is the case during infection with hypervirulent *Mtb* strains^35^. Abrogation of Type I responses reverses this trend and is being considered as a host-directed therapy for TB^36^. Accordingly, the expression of Type I genes was significantly higher in BAL samples obtained 3 weeks after vaccination from animals that received BCG, and significantly lower in animals that received MtbΔ*sigH* (Fig. 3f). The IRF2 gene expression level was lower in the BAL of BCG-vaccinated and more than twofold higher in the BAL of MtbΔ*sigH*-vaccinated animals (Fig. 3f). The expression of prototypical Type I molecule IFN-γ was also higher in BMDMs infected with BCG and *Mtb*, relative to those infected with MtbΔ*sigH* (Fig. 3c).

### MtbΔ*sigH* induces protective immune signatures in the lung

**Figure 4:**
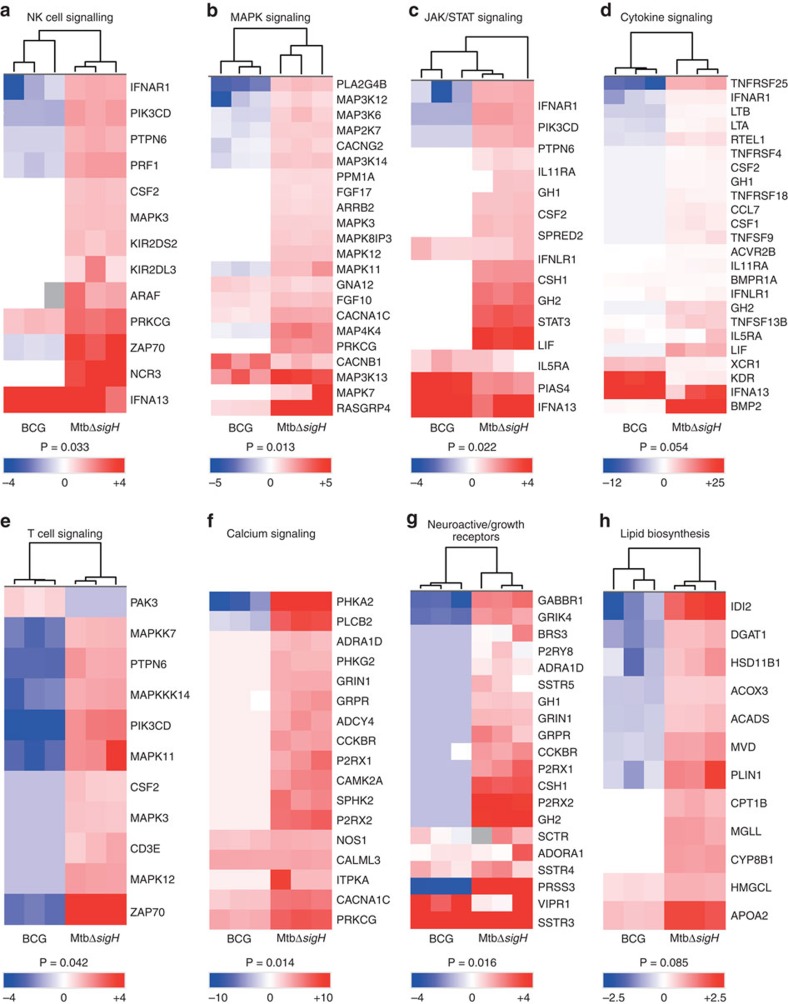
Transcriptomics from BAL 3 weeks after immunization. Total RNA isolated from BAL samples of three animals vaccinated with BCG and another three with MtbΔ*sigH*, obtained 3 weeks after vaccination, was subjected to amplification and macaque-specific DNA microarray analysis. The expression of genes belonging to natural killer cells (**a**), MAP Kinase (**b**), JAK/STAT (**c**), cytokine (**d**), T cells (**e**), calcium signalling (**f**), neuroactive/growth receptors (**g**) and lipid biosynthesis (**h**) pathways were induced to significantly higher levels in the BAL of animals vaccinated with MtbΔ*sigH*, relative to those vaccinated with BCG. *P* values shown are derived from the analysis of significant terms in DAVID with Bonferroni correction for multiple comparisons. BAL samples from three animals in each group were used for microarray experiments.

Global immune responses to vaccination with MtbΔ*sigH* or BCG were analysed by transcriptome profiling of BAL samples collected before vaccination and 3 weeks post vaccination. The genes significantly induced in BAL after BCG vaccination were involved in cellular transport, DNA binding by regulatory protein, RNA processing or were part of the lumen, non-membrane-bound organelle or macromolecular complex assembly (Fig. 4). The only major category of genes induced following BCG vaccination was that categorized as pertaining to immune system development (Fig. 4). Conversely, the expression of genes involved in NK cell signalling, MAPK signalling and JAK/STAT signalling, cytokine signalling, T-cell signalling, calcium signalling, neuroactive/growth receptors and lipid biosynthesis were induced to higher levels in the BAL from MtbΔ*sigH*-vaccinated macaques (Fig. 4). Further, a large majority of these genes were either not expressed or were expressed to significantly lower levels in the BAL of animals vaccinated with BCG (Fig. 4). Thus, vaccination with MtbΔ*sigH* appeared to result in the induction of a markedly stronger innate immune response, as indicated by the differential induction of NK cell, MAPK and JAK/STAT signalling pathways as well as genes from the cytokine, T-cell receptor and calcium signalling pathways (Fig. 4), although the differential persistence of the two mycobacterial strains could have played a role in this differential response. In addition, enhanced immune cell differentiation, proliferation, activation and processing, as well as macromolecular synthesis, which are associated with heightened cytokine and T-cell responses, were evidenced by the increased expression levels of neuroactive/growth factor receptor signalling, potentiation and lipid biosynthesis pathways in BAL samples derived from animals vaccinated with MtbΔ*sigH* (Fig. 4).

### Local increases in T cells due to MtbΔ*sigH* vaccination

**Figure 5:**
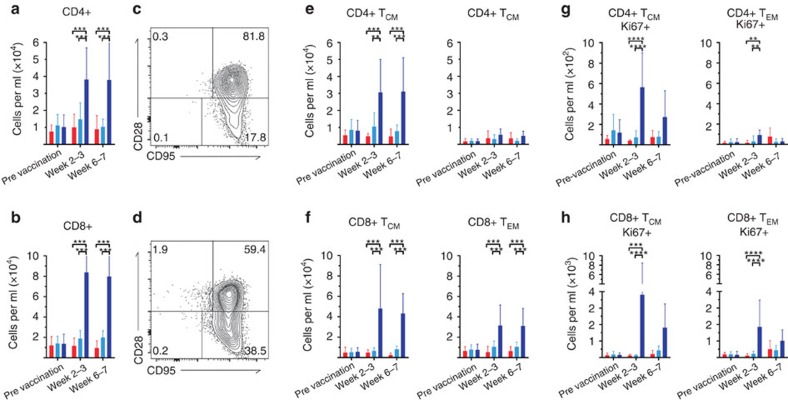
T-cell response to immunization in BAL. Vaccination with MtbΔ*sigH* (dark blue) induced a significantly higher central memory immune response relative to BCG vaccination (light blue), or no vaccination (red). Quantification of CD4^+^ (**a**) and CD8^+^ (**b**) T cells migrating to the lung after vaccination. Representative plots of central memory (CD28^+^CD95^+^), effector memory (CD28^−^CD95^+^) and naive (CD28^+^CD95^−^) CD4^+^ (**c**,**e**) and CD8^+^ (**d**,**f**) T cells in BAL. Quantification of CD4^+^ (**e**) T_CM_ and T_EM_ cells, CD8^+^ T_CM_ and T_EM_ cells (**f**) in BAL, and the proliferative capability of these cells (**g**,**h**). *P<0.05; **P<0.01; ***P<0.001; ****P<0.0001 using two-way ANOVA with Tukey’s correction for multiple comparisons. Data are means±s.d. BAL samples from all 21 animals (*n*=7, three groups) were included in the flow cytometry experiments and analyses. Samples from all seven animals in each group were used for analysis at each time point.

Local and systemic immune responses were compared following vaccination by analysis of BAL and blood, respectively. Marked increases in CD4^+^ and CD8^+^ T-cell numbers were found in BAL immediately post vaccination (*P*<0.001; two-way analysis of variance analysis of variance (ANOVA) with Tukey’s correction; Fig. 5a,b); however, no differences in the frequency of CD4^+^ and CD8^+^ T cells were observed in the periphery post vaccination (Supplementary Fig. 2). In addition, no differences in the chemokine receptors CXCR3, CXCR4, CCR7 or activation marker CD69 were detected systemically, which indicated no significant variation in the functional phenotype of T cells in the blood (Supplementary Fig. 3). However, a significant increase in the number of circulating CD4^+^CCR5^+^ T cells was discovered in the MtbΔ*sigH* group between weeks 2 and 3 (*P*<0.05; two-way ANOVA with Tukey’s correction; Supplementary Fig. 3). No differences were observed in the memory status of circulating T cells, including central (T_CM−_CD28^+^CD95^+^) and effector memory (T_EM−_CD28^−^CD95^+^) cells or naïve T cells (CD28^+^CD95^−^; Supplementary Fig. 2)^37^.

**Figure 6:**
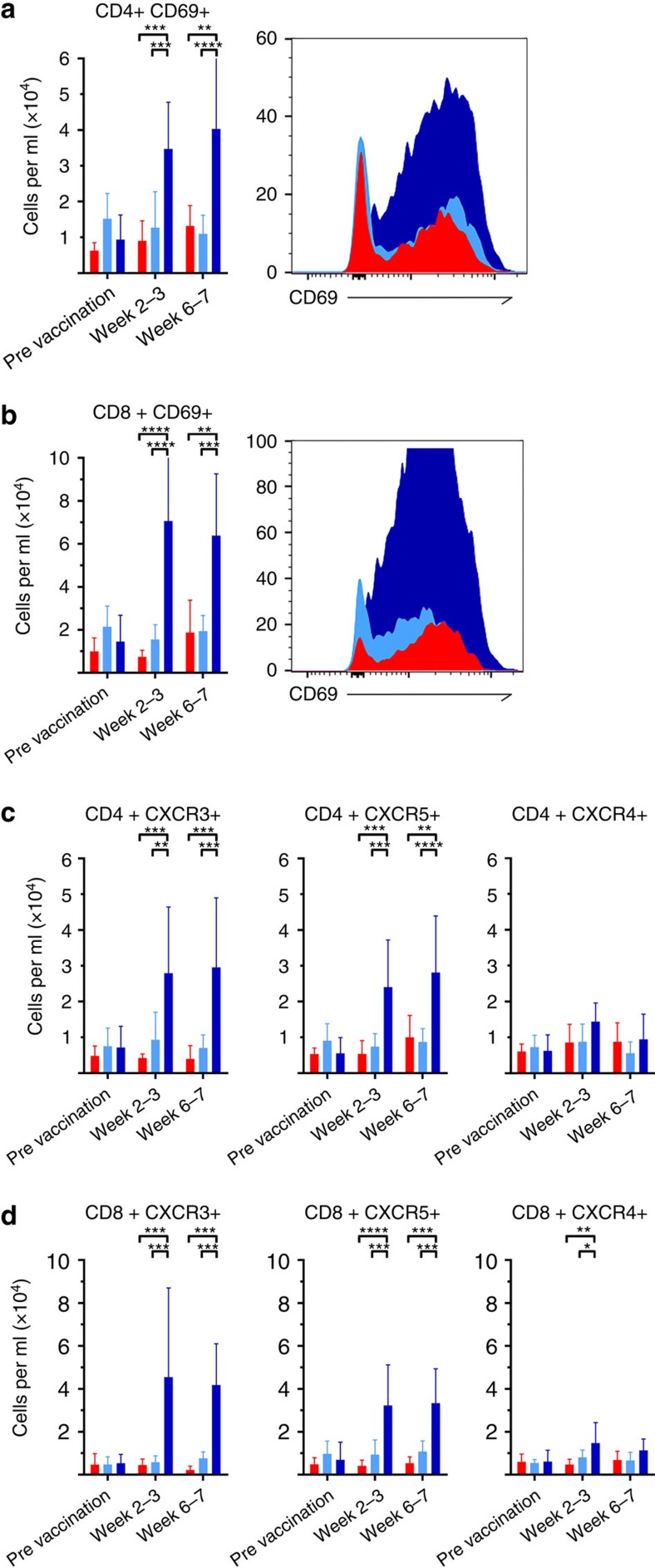
Local T-cell phenotype to immunization in BAL. Vaccination with MtbΔ*sigH* (dark blue) induced a significantly stronger T_H_1 cell response relative to BCG vaccination (light blue), or no vaccination (red). Quantification of and representative histograms of CD4^+^CD69^+^ (**a**) and CD8^+^CD69^+^ (**b**) T cells migrating to the lung after vaccination. Absolute cell counts of phenotypic markers CXCR3, CCR5 and CXCR4 in CD4^+^ (**c**) and CD8^+^ (**d**) T cells in BAL at different stages of infection. *P<0.05, **P<0.01, ***P<0.001, ****P<0.0001 using two-way ANOVA with Tukey’s correction for multiple comparisons. Data are means±s.d. BAL samples from all 21 animals (*n*=7, three groups) were included in the flow cytometry experiments and analyses. Samples from all seven animals in each group were used for analysis at each time point.

While no significant changes were discovered in T-cell frequencies in the peripheral blood, a strong lung-specific central and effector memory response was initiated post vaccination with MtbΔ*sigH*. This response was apparent immediately after vaccination by significant increases in the number of CD4^+^ central memory (T_CM_) (*P*<0.01–0.001; two-way ANOVA with Tukey’s correction) as well as CD8^+^ T_CM_ and effector memory (T_EM_; *P*<0.001) in the BAL (Fig. 5c–f), with a marked increase in proliferation as measured by Ki67 positivity (*P*<0.01–0.0001; Fig. 5g,h). A significant increase in the number of CD69^+^ T cells (*P*<0.001 for MtbΔ*sigH* versus BCG and *P*<0.01–0.001 for MtbΔ*sigH* versus unvaccinated groups; Fig. 6a,b) indicated that T cells in BAL were antigen-stimulated via the T-cell receptor. The polarity of the T-cell response was examined using the markers CCR5 and CXCR3, which are preferentially expressed by Th1 cells, as well as CXCR4, which is preferentially expressed by T-helper 2 (Th2) cells. The results revealed that T cells in BAL immediately post-MtbΔ*sigH* vaccination preferentially expressed high levels of CCR5 and CXCR3 compared with T cells recruited following vaccination with BCG (*P*<0.01–0.001; Fig. 6c,d). Thus, aerosol vaccination with MtbΔ*sigH* induced transcriptomic and cellular signatures indicative of a strong Th1 response that resulted in accumulation of memory T cells.

### Vaccination with MtbΔ*sigH* protects from lethal *Mtb* challenge

To evaluate the potential of the attenuated mycobacterium to serve as a TB vaccine, all animals were challenged with a high dose of *Mtb* CDC1551, which has historically produced lethal TB in rhesus macaques within 10 weeks^20^,^32^. Unvaccinated animals rapidly developed pulmonary granulomatous pathology with rapid increases in body temperatures (*P*<0.01 for MtbΔ*sigH* relative to BCG and *P*<0.0001 for MtbΔ*sigH* versus unvaccinated groups; two-way ANOVA with Tukey’s correction) and serum CRP levels (*P*<0.0001 for MtbΔ*sigH* versus both other groups; Fig. 2b,d), as well as a swift decline in body weights *P*<0.01 for MtbΔ*sigH* relative to BCG and *P*<0.0001 for MtbΔ*sigH* versus unvaccinated groups; Fig. 2c). Control animals also exhibited high levels of pulmonary granulomatous involvement by radiology (Fig. 2e). The same clinical measure also increased, albeit to a lesser degree, in animals that were aerosol-vaccinated with BCG, while animals vaccinated with MtbΔ*sigH* exhibited virtually no evidence of disease (*P*<0.0001 for MtbΔ*sigH* versus unvaccinated as well as versus BCG; Fig. 2b–e). Three weeks post-*Mtb* challenge (week 11), the increase in body temperatures in unvaccinated animals was significant relative to BCG-vaccinated (*P*<0.01) and highly significant relative to MtbΔ*sigH*-vaccinated animals (*P*<0.0001; two-way ANOVA with Tukey’s correction; Fig. 2b). Five weeks post challenge (week 13), the differences in body temperature between the unvaccinated and BCG-vaccinated groups were significant (*P*<0.05); the differences between the BCG and MtbΔ*sigH*-vaccinated groups were very significant (*P*<0.01); and the differences between the unvaccinated and MtbΔ*sigH*-vaccinated groups were highly significant (*P*<0.0001; Fig. 2b). The differences in body weight between the unvaccinated and MtbΔ*sigH*-vaccinated groups and the BCG and MtbΔ*sigH*-vaccinated groups were very significant at week 11 (*P*<0.01; Fig. 2c) and while by week 13, these differences had become highly significant (*P*<0.0001; two-way ANOVA with Tukey’s correction). Profoundly lower serum CRP levels were detected in the macaques vaccinated with MtbΔ*sigH* relative to macaques that were not vaccinated at weeks 11 and 13 and at time of euthanasia (*P*<0.0001). At both week 11 (*P*<0.05) and while euthanasia (*P*<0.0001; two-way ANOVA with Tukey’s correction), the serum CRP levels detected in the macaques vaccinated with MtbΔ*sigH* were also significantly lower than levels measured in the BCG-vaccinated group (Fig. 2d). Similarly, animals vaccinated with MtbΔ*sigH* exhibited significantly lower chest X-ray (CXR) scores, consistent with lack of disease following lethal challenge, in this group, relative to either BCG-vaccinated or unvaccinated macaques (*P*<0.0001 in both cases). While extensive pathology was not discernable in the lungs of macaques vaccinated with the mutant, a majority of those vaccinated with BCG exhibited moderate CXR scores, again significantly lower than those in the unvaccinated group, where pathology consistent with military TB could be observed in a majority of animals.

### Vaccination with MtbΔ*sigH* reduces *in vivo* bacterial burdens

**Figure 7:**
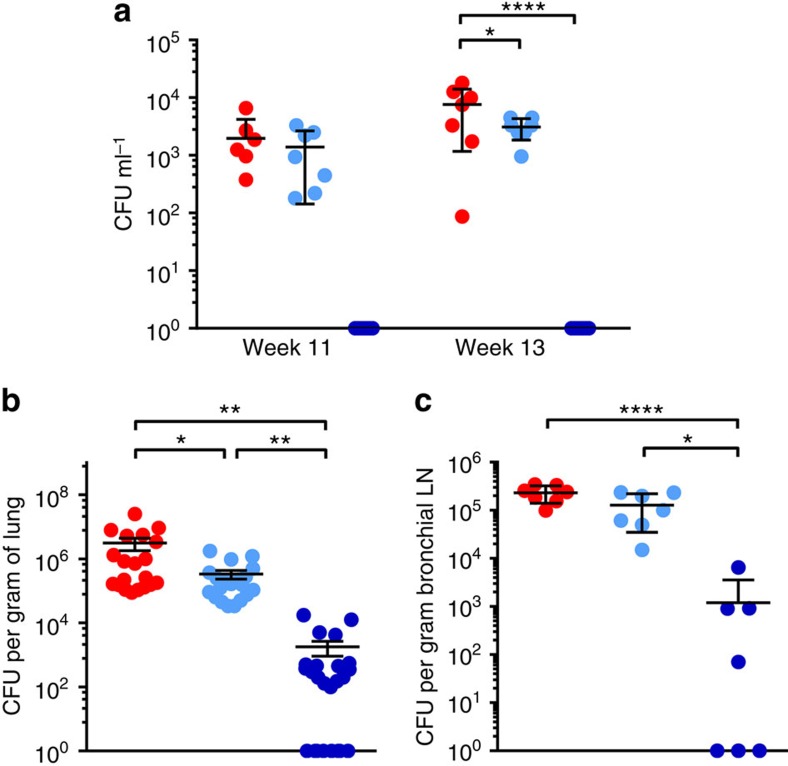
Bacterial burden following lethal *Mtb* challenge. (**a**) *Mtb* CFU levels in total BAL samples at week 11 (3 weeks after challenge) and week 13 (5 weeks after challenge; (**b**). *Mtb* levels per gram of lung tissue at necropsy (**c**). *Mtb* burdens per gram of bronchial lymph node tissue at *P<0.05; **P<0.01; ***P<0.001; ****P<0.0001, (**a**) two-way ANOVA and (**b**,**c**) one-way ANOVA with Tukey’s multiple testing correction. Data are means±s.d. Samples from all seven animals in each group were used for analysis at each time point. For analysis of lung burdens, four pooled samples (two each from left and right lung) from each macaque, each representing five distinct lung sections were used (**b**). For analysis of bronchial lymph node burdens, one section from each animal was analysed.

The protection conferred by MtbΔ*sigH* was also evident following evaluation of bacterial burdens. Significantly lower Mtb was recovered from BAL from MtbΔ*sigH*-vaccinated animals relative to those vaccinated with BCG at 3 and 5 weeks after challenge (weeks 11 and 13; Fig. 7a). At the time of euthanasia, the total lung burden in animals vaccinated with MtbΔ*sigH* was three logs lower than burdens in unvaccinated (*P*<0.01) and two logs lower than burdens in BCG-vaccinated (*P*<0.01) animals, while bacterial loads in animals vaccinated with BCG were only 0.5–1 logs lower than in the control group (*P*<0.05; one-way ANOVA with Tukey’s correction; Fig. 7b). We were unable to culture *Mtb* from >42% of all lung sections obtained from macaques vaccinated with MtbΔ*sigH*. In contrast, every section obtained from either unvaccinated animals or those vaccinated with BCG was positive for *Mtb*. Thus, the *Mtb* burden was significantly lower in the lungs of MtbΔ*sigH*-vaccinated animals following lethal challenge compared with those vaccinated with BCG. Similar results were obtained when bronchial lymph node bacterial burdens were analysed at necropsy (Fig. 7c). Using a combination of hygromycin resistance/sensitivity and PCR, we verified that the obtained CFUs were *Mtb* and not residual MtbΔ*sigH* or BCG.

### Vaccination with MtbΔ*sigH* leads to reduced lung pathology

**Figure 8:**
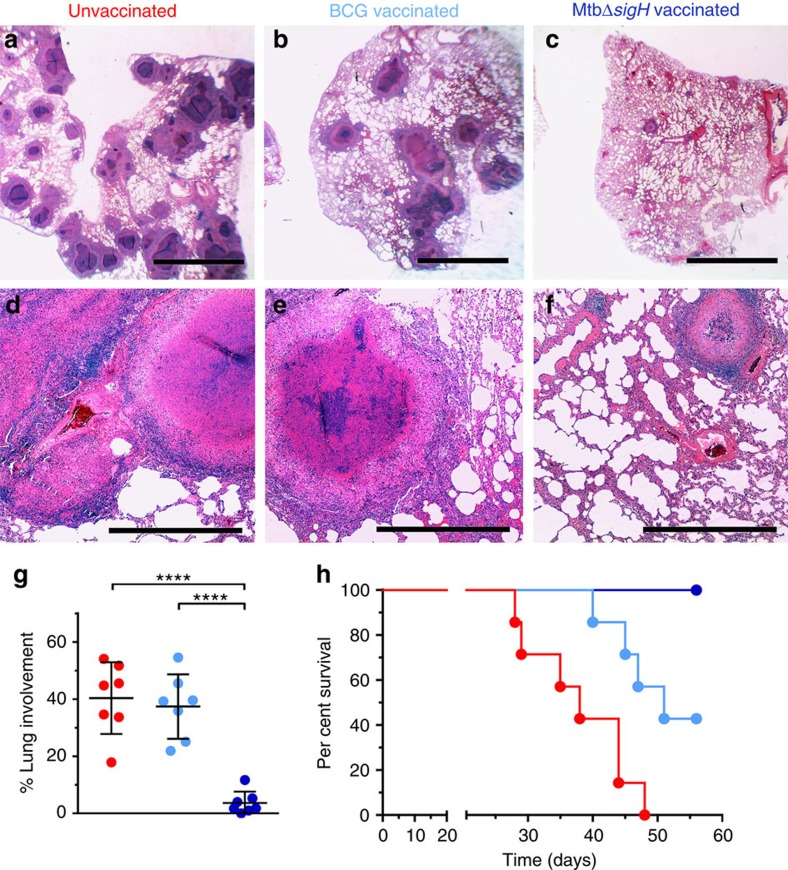
Histopathological and survival analysis of lungs of *Mtb*-infected animals. Haematoxylin and eosin (H&E) staining from representative animals in each of the three groups. (**a**,**d**) Vaccine-naive group with miliary, white 2–4 mm granulomas and scattered lobular multicoloured areas of consolidation. (**b**,**e**) BCG-vaccinated with localized dark red lobar pneumonia and (**c**,**f**) MtbΔ*sigH*-vaccinated with no apparent gross lesions. Black scale bars in (**a**–**c**), 5 mm and (**d**–**f**), 500 μm. (**g**) Morphometric measures of pulmonary pathology in the different groups of unvaccinated (red), BCG-vaccinated (light blue) and MtbΔ*sigH*-vaccinated (dark blue) animals. ****P<0.0001 with (**g**) one-way ANOVA using Tukey’s multiple testing correction. Data are means±s.d. (**h**) Survival proportion Kaplan–Meier curves for the three groups of animals, using Mantel–Cox (log-rank) survival analysis. At least three systematic random microscopic fields from each lung, representing most lung lobes, from each of the animals in every group were used for morphometric analysis (**g**). Data from each of the seven animals per group was used for survival proportions analysis (**h**).

The results of the pulmonary pathology analyses mirrored those obtained following analysis of bacterial burdens. Animals vaccinated with MtbΔ*sigH* exhibited significantly fewer pulmonary lesions on challenge, as determined by both gross and histopathological examination (Fig. 8a–f and Supplementary Fig. 4) and morphometric quantitation (Fig. 8g). Thus, the animals vaccinated with MtbΔ*sigH* had fewer granulomas (the extent of lung affected by TB lesions following *Mtb* infection encompassed an average of 4%), and less TB-related pathology (for example, oedema, pneumonia and generalized foci of inflammation) than animals in the other two groups (where ∼40% of the lung was affected; *P*<0.0001 in both cases; one-way ANOVA with Tukey’s correction; Fig. 8g). The clinical and microbiological differences also correlated with significant differences in overall survival rates following challenge (Fig. 8h). All seven unvaccinated animals succumbed to massive pulmonary TB following a lethal aerosol *Mtb* challenge within a median of 36 days. Five out of seven BCG-vaccinated animals also succumbed to TB, within a median of 48 days. None of the MtbΔ*sigH*-vaccinated animals exhibited any overt signs of disease or required euthanasia (Fig. 8h).

### Differential T-cell responses in animals post challenge

To assess an immunologic basis for protection induced by MtbΔ*sigH*, the functional phenotype of circulating CD4^+^ and CD8^+^ T cells was analysed by employing the same markers used for evaluating pulmonary responses following vaccination. No significant differences in the percentages of CD4^+^ and CD8^+^ central or effector memory cells were observed in peripheral blood immediately following challenge (Supplementary Fig. 2). However, a significant increase in CD4^+^ CCR5^+^ T cells was identified 7 weeks after *Mtb* challenge, as well as a significant decrease in CD8^+^ CXCR3^+^ T cells in peripheral blood at weeks 3 and 7 following challenge in unvaccinated animals (Supplementary Fig. 3).

**Figure 9:**
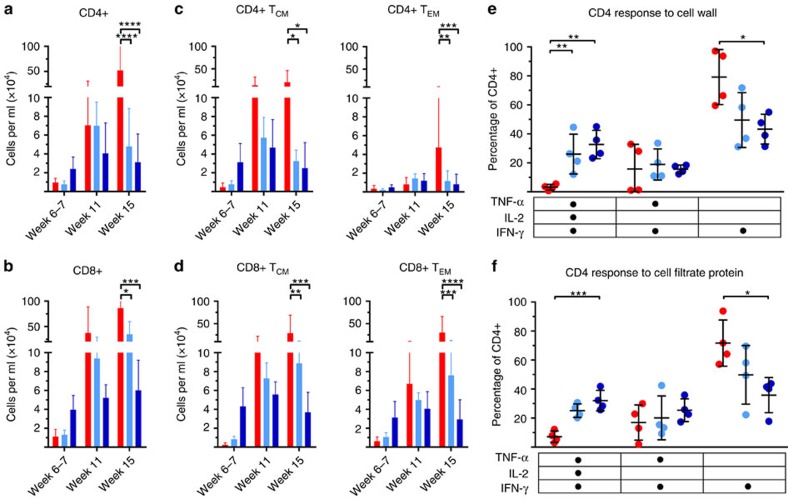
Immune responses in the lung post-*Mtb* challenge. (**a**,**b**) Quantification of cells per ml of BAL of (**a**) CD4^+^ and (**b**) CD8^+^ T cells and of (**c**) CD4^+^ T_CM_ and T_EM_ cells and of (**d**) CD8^+^ T_CM_ and T_EM_ cells migrating to the lung after challenge in groups of unvaccinated (red), BCG-vaccinated (light blue) and MtbΔ*sigH*-vaccinated (dark blue) animals. (**e**,**f**) Percentage of CD4 T cells from PBMCs prepared from whole blood, producing gamma interferon, interleukin 2 and tumour necrosis factor alpha in response to (**e**) the *Mtb* cell wall and to (**f**) *Mtb* cell filtrate protein. *P<0.05, **P<0.01, ***P<0.001, ****P<0.0001 using two-way ANOVA with Tukey’s correction for multiple comparisons. Data are means±s.d. BAL samples from all 21 animals (*n*=7, three groups) were included in the flow cytometry experiments and analyses (**a**–**d**). For antigen-specific studies, samples from four animals per group were employed (**e**–**d**).

Consistent with overall lung involvement and chest radiograph scores, a significantly greater number of CD4^+^ and CD8^+^ T cells including T_CM_ and T_EM_ were present in BAL in the unvaccinated animals compared with both BCG and MtbΔ*sigH*-vaccinated animals (Fig. 9a–d). The increased numbers of T cells in BAL in unvaccinated animals were also evident from representative histograms of CD69^+^ T cells (Supplementary Fig. 5a,b). While no significant differences were observed in CXCR3, CCR5 and CXCR4 expression by CD4^+^ or CD8^+^ T cells between the two vaccinated groups (Supplementary Fig. 5c,d), a significant decrease in the number of CD8^+^CXCR4^+^ was noted at weeks 11 and 15 in the MtbΔ*sigH*-vaccinated group compared with the other two groups (Supplementary Fig. 5d).

### Comparison of polyfunctional *Mtb*-specific T-cell responses

To examine antigen-specific responses to *Mtb*, isolated peripheral blood mononuclear cells (PBMCs) collected 5 weeks post challenge were stimulated with whole *Mtb* cell wall and cell filtrate protein, and responses were analysed using intracellular cytokine staining for IFN-γ, IL-2 and TNF-α. The results indicated that vaccination with MtbΔ*sigH* and BCG correlated with significant increases in the percentage of polyfunctional, IFN-γ^+^, IL-2^+^, TNF-α^+^ CD4^+^ T cells, whereas unvaccinated animals displayed a significantly greater number percentage of monofunctional CD4^+^ T cells (Fig. 9e,f). Unfortunately, samples from other time points were not available for analysis.

### Profound iBALT post challenge in MtbΔ*sigH*-vaccinated animals

**Figure 10:**
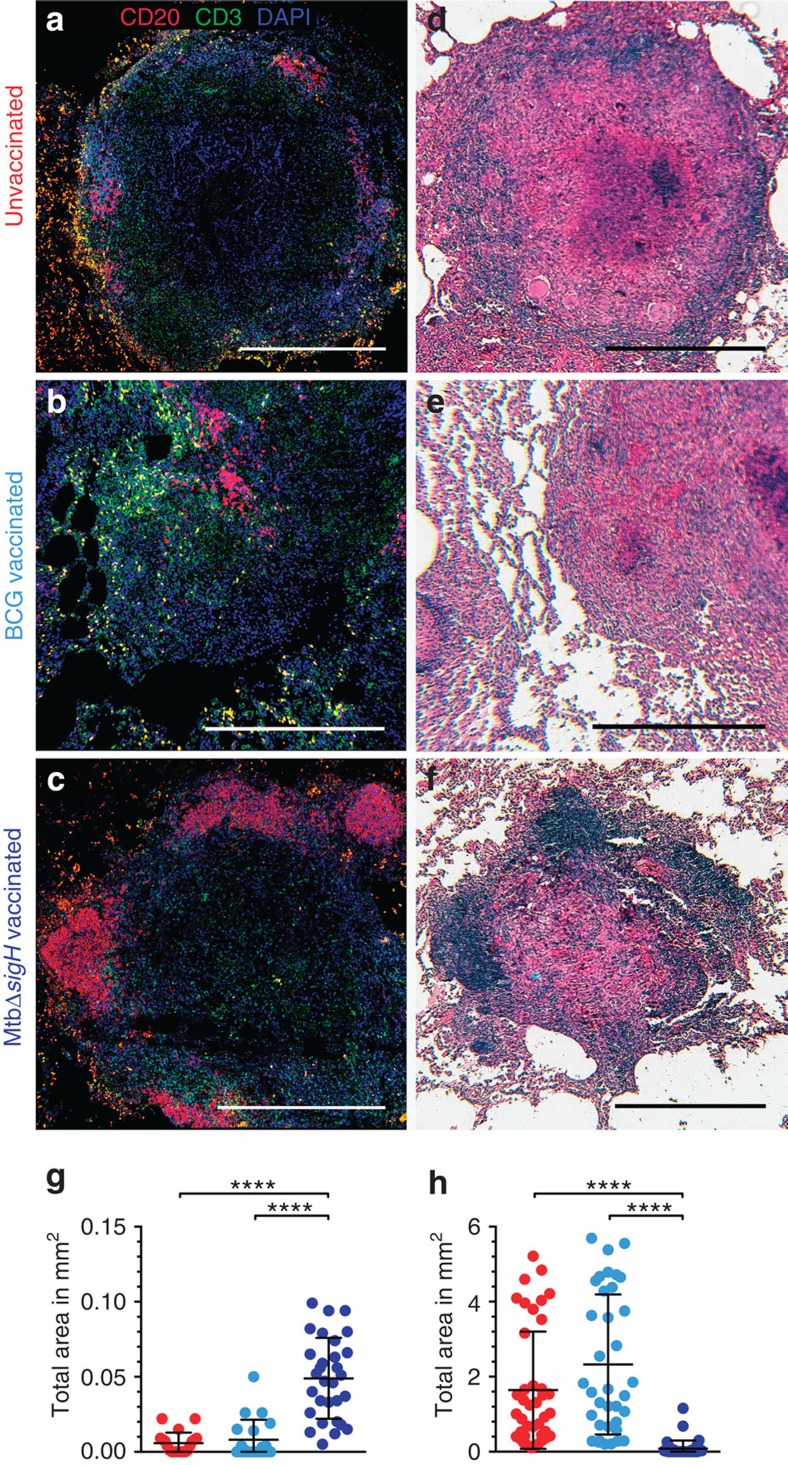
Induction of BALT after lethal *Mtb* challenge correlates with protection from pulmonary granulomatous TB. (**a**–**c**) Co-staining of lung sections with CD3 (green) and CD20 (red) and staining of corresponding sections (**d**–**f**) with H&E revealed that (**a**,**d**) vaccine-naive animals and (**b**,**e**) BCG-vaccinated animals had significantly reduced iBALT follicle formation in comparison with (**c**,**f**) MtbΔ*sigH*-vaccinated animals, at necropsy, after lethal *Mtb* challenge. White and black scale bars, 500 μm. Quantification of multiple lesions in six different animals were used the analysis. (**g**) Total area (mm^2^) of iBALT follicles and (**h**) granulomatous pathology in unvaccinated (red), BCG-vaccinated (light blue) and MtbΔ*sigH*-vaccinated (dark blue) animals. ****P<0.0001 using Student’s *t*-test. Data are means±s.d. At least 10 sections from each slide derived from a lung block from multiple animals in each group were used for statistical analysis (**g**–**h**).

Lung samples collected after lethal *Mtb* challenge from unvaccinated (Fig. 10a,d), BCG-vaccinated (Fig. 10b,e) and MtbΔ*sigH*-vaccinated (Fig. 10c,f) macaques at the time of necropsy were assayed for iBALT by histopathology and immunofluorescence with CD3 and CD20 followed by confocal microscopy and image analysis. We observed that protection in each of these groups of macaques was strongly associated with levels of iBALT. Thus, the few, small granulomas in the lungs of animals vaccinated with MtbΔ*sigH* and challenged with *Mtb* were characterized by the presence of multiple well-organized iBALT per lesion (Fig. 10c). The presence of the follicles was also apparent in the cognate haematoxylin and eosin stains (Fig. 10 c,f). These follicles were, in general, associated with granulomas and appeared to be an outgrowth of the outer lymphocyte-rich layer (Fig. 10 c,f). While B cells constituted the majority in these follicles, CD3^+^ cells were also present (Fig. 10c). In contrast to the multiple well-developed iBALT associated with granulomas in MtbΔ*sigH*-immunized animal, animals from the other groups (Fig. 10a,b,d,e) had fewer iBALT that were less well organized. The total area of iBALT in MtbΔ*sigH*-vaccinated macaques was significantly greater after *Mtb* challenge (Fig. 10g) than in either of the other groups, despite the fact that the area occupied by granulomas in the MtbΔ*sigH*-vaccinated group was significantly less compared with the other two groups after *Mtb* challenge (Fig. 10h). This further emphasizes the difference in BALT induction and the correlation between iBALT and latent control of *Mtb* infection.

## Discussion

Previously, we showed that aerosolization of a high dose of MtbΔ*sigH* into the lungs of rhesus macaques resulted in nonpathogenic infection^20^. Here we demonstrate that pulmonary vaccination with this mutant protected against lethal challenge in macaques via the induction of a potent memory T-cell response. Further, we establish a strong correlation between the latent control of infection and iBALT, suggesting that these lymphoid structures facilitate control of TB by maintaining LTBI or sterilizing infection. Induction of BALT in response to respiratory infections is known to not only result in protection, but such responses are also less pathologic^38^. Secondary T_FH_ cells, which constitute the great majority of lymphoid follicle (including iBALT) T cells, expand from memory T cells in a B-cell-aided manner^39^. Our results therefore suggest that the interplay between B and T cells is critical for the proper development of granulomatous-protective responses to TB. This study substantiates previous work using the macaque model of TB, which showed that B cells produced antibody and were important for control of TB^40^. Our results suggest that future studies should unravel the function of B cells recruited to the lung to understand immunity from TB.

While the correlates of vaccine-induced protective immunity against TB are not completely understood, T lymphocytes expressing markers of central memory response (dual positivity for CD28 and CD95)^37^ are critical for protection. A model of protective immunity that is currently gaining credence suggests that initial vaccination(s) resulting in a spike in effector response can result in the establishment of a central memory response, which is likely to result in long-term protection^41^. T_CM_ are extremely proliferative and can rapidly evolve into large numbers of effector cells expressing high levels of pro-inflammatory cytokines (for example, IFN−γ). Consequently, the finding that pulmonary vaccination with MtbΔ*sigH* resulted in potent T_CM_ (as well as T_EM_, particularly CD8^+^) responses during the post-vaccination phase that corresponded to a strong T_EM_ response in the challenge phase merits further evaluation. The role of chemokine receptors, including CXCR3, is critical to the recruitment of CD8^+^ T cells, and CXCR3 expression on T_CM_ is strongly correlated with effective anti-pathogen memory responses^42^. BAL transcriptome profiling results indicated that animals vaccinated with MtbΔ*sigH* developed a highly productive pulmonary immune response, both in terms of magnitude and breadth. Prior studies have established that CD4^+^ T cells with specificity for mycobacterial antigens are critical for the control of *Mtb* infection^43^, and that CD8^+^ T cells play an increasingly important role in this process^41^,^44^. SigH induces pathogen antioxidant responses during stress^45^,^46^. MtbΔ*sigH* is unable to induce expression of the transcriptionally linked thioredoxins and thiol peroxidases^45^ that act as antioxidant buffers against the host oxidative burst^47^. Consequently, the *antioxidant* response of the MtbΔ*sigH* mutant was strongly crippled^47^, and the resulting production of oxidants likely enhanced antigen-specific CD8^+^ T-cell responses through the cross-priming of antigen presentation^48^. In addition, the ability of MtbΔ*sigH* to persist longer than BCG allowed for increased stimulation and antigen presentation, inducing a stronger, more robust, T-cell repertoire^49^. Further characterization of this T_CM_ response is however necessary to substantiate that these cells are not long-lived T_EM_ cells, since MtbΔ*sigH* antigens persist longer than BCG.

The ability of aerosol MtbΔ*sigH* vaccination to elicit highly protective responses in macaques could have roots in the fundamental processes that govern *Mtb*–macrophage interactions. *In vitro*, macrophages infected with this mutant elicited greater expression of Th1 pro-inflammatory cytokines TNF-α and IL-1β, and lower Type I interferon, relative to those infected with *Mtb* and BCG (this study and ref. 22). Together, these results indicate that the interaction of MtbΔ*sigH* with host phagocytes is critically altered by the loss of the bacillus’s ability to induce SigH-mediated antioxidant functions, relative to BCG. TNF can restrict macrophage-contained *Mtb* by several mechanisms, including by potently inducing early inflammatory responses^50^, transducing apoptotic signals through activation of caspase 8 (ref. 51) activating CD8 cells^52^ and potentiating IFN-γ-induced killing of *Mtb*^53^,^54^. On the other hand, BCG induces strong expression of the SigH regulon (including antioxidants) and is therefore unable to alter these interactions. In fact most BCG strains exhibit duplication of *sigH*, likely rendering it even less susceptible to phagocyte oxidative mechanisms^55^. *In vitro* results thus hint at the mechanism by which pulmonary vaccination with MtbΔ*sigH* could generate protective responses. Unable to counter the host oxidative burst, the mutant elicited greater IL-1β and TNF-α expression leading to significantly greater antimicrobial response, apoptosis and antigen presentation. That *Mtb* uses Type I signalling to modulate IL-1β expression is well established^56^.

CD4^+^ T cells play a key role in the control of *Mtb* infection, and polyfunctional cytokine production of antigen-specific Th1 cells is associated with this control^57^. Animals vaccinated with MtbΔ*sigH* displayed higher frequencies of *Mtb* cell wall- and culture filtrate protein-responsive polyfunctional CD4^+^ T cells compared with unvaccinated animals. Recent data have shown that markers of T-cell immune activation in human TB are associated with smear and culture conversion during anti-TB treatment^58^. Nonetheless, the current study had some limitations; for example, protection was only demonstrated in a homologous strain. Thus, future experiments should test the efficacy of the current and related mutants against high-virulence heterologous strains of the same clade as CDC1551, as well as of a different lineage. Second, a lethal challenge at 8 weeks post vaccination may not sufficiently model protection arising from long-term antigen-specific memory T-cell responses. A prior report suggested that BCG failed to induce long-term central memory responses to *Mtb* infection, which may account for its failure to protect adults against TB^10^. Successful vaccination against TB will require elicitation of both a stable and broad repertoire of memory responses to ensure effective protection against the pathogen during a subsequent infection^41^. While the current results did not establish long-term immunity based on effective elicitation of T_CM_ responses, the findings did demonstrate that prior pulmonary vaccination with MtbΔ*sigH* resulted in a markedly superior elicitation of both CD4^+^ and CD8^+^ T_CM_ responses relative to BCG vaccination. An abundance of data has indicated that T_CM_ lymphocytes retain a potent proliferative capacity and can differentiate into the T_EM_ phenotype when re-exposed to the cognate antigen^59^. The current data demonstrated that MtbΔ*sigH* elicited a more efficient post-vaccination T_CM_ response, a greater T_EM_ response following challenge and conferred stronger protection. In this study, vaccines were delivered via aerosol, although BCG is given to recipients intradermally. It is impossible to directly compare the results from the aerosol–BCG group to the effectiveness of intradermal BCG vaccination. There have been recent attempts, however, to deliver vaccines, directly to the lung^11^, driven by the pioneering work of Barclay *et al*.^12^, who showed that macaques could be protected against TB by aerosolized BCG. While a direct comparison of the effectiveness of aerosol-to-intradermal-administered BCG was beyond the scope of this study, preliminary data and prior reports of intradermal BCG vaccination^14^,^24^,^60^ suggest that aerosol–BCG outperformed intradermal vaccination in macaques.

Although BCG induces potent cellular responses in infants and protects them, neither the responses nor the efficacy are generally considered to be long lasting^61^, although some reports strongly suggest that this is possible^9^. Furthermore, BCG does not completely protect against pulmonary TB including reactivation and reinfection^61^. New vaccines against TB are therefore urgently needed. It has therefore been hypothesized that rationally attenuated strains of *Mtb* are likely better at serving as vaccine platforms against TB, since these strains are human-adapted, in contrast to bovine-adapted BCG^61^. There are other issues that limit the effectiveness of BCG. It is only able to induce cytokines associated with long-lasting control of *Mtb* infection several months post vaccination, meanwhile enabling a window of dissemination^62^. Furthermore, *Mtb*, unlike BCG, retains known immunodominant epitopes for humans. Finally, it has been argued that *Mtb* expresses genes important for the evasion of host immune responses. SigH is one such gene allowing the pathogen to significantly induce the expression of thioredoxins, which attenuate phagocyte oxidative burst^20^,^22^,^47^. Thus, MtbΔ*sigH*, a human-adapted strain with a significant immune-evasion impairment, is exceedingly safe even after direct high-dose delivery into primate lungs.

In summary, our results provide a roadmap for identifying correlates of protection from TB in a highly human-like model. While it remains to be seen whether such responses can also be elicited following intradermal vaccination, there is considerable renewal of interest in matching the route of vaccination with that of infection in generating protection against TB^13^.

## Methods

### Nonhuman primates

All animal procedures were approved by the Institutional Animal Care and Use Committee and were performed in strict accordance with the NIH guidelines. Twenty-one specific-pathogen-free, retrovirus-free, mycobacteria-naive, adult rhesus macaques, bred and housed at the Tulane National Primate Research Centre (TNPRC), between the ages of 3 and 12 years were assigned to three groups of seven on the basis of power calculations, which suggested that statistical significance could be detected with sufficient power in these group sizes if addressing a reduction in the average lung bacterial burden by 1log. One group of macaques (*n*=7) remained unvaccinated, a second group (*n*=7) was vaccinated with a target dose of 1,000 CFU of *M. bovis* BCG (Danish) while the third group (*n*=7) was vaccinated with an equivalent dose of the MtbΔ*sigH* isogenic mutant in the CDC1551 background. Aerosol vaccination was conducted using the same equipment and procedural configuration as the *Mtb* challenge component of the study (see below). The average ages of the animals within each group were 7.42±4.26 years for the unvaccinated group, 7.38±4.50 years for the BCG-vaccinated group and 6.75±4.02 years for the MtbΔ*sigH* group, and these were not statistically significant differences (Supplementary Fig. 6).

### Aerosol procedures for both vaccination and *Mtb* challenge

Animals were both aerosol-vaccinated and challenged with *Mtb* using the same methodology and equipment configuration. A custom head-only dynamic inhalation system housed within a class III biological safety cabinet was used for this purpose^63^. The use of this inhalation system for aerosol delivery to non human primate (NHP)^63^ has been described for numerous studies involving *Mtb*^20^,^25^,^29^,^32^ as well as other agents^64^. Initially, the respective microbial efficiencies of MtbΔ*sigH* gene mutant and the parental *Mtb* CDC1551 strains were determined through a series of aerosol-only studies. The microbial efficiencies were used to estimate achievable aerosol doses that could be delivered to each animal using mathematical formula catered to this purpose^65^ for both the aerosol vaccination and subsequent challenge experiments. The animal vaccinations (and challenge exposures) were performed singly, and a ‘target’ dose is reported for the group based on prevailing experimental conditions, including individual animal respiratory rate, during each exposure event. Actual individual dose is based on *post hoc* analysis of active aerosol sampling of the inhalation chamber during the time of each vaccination and bacterial challenge. All aerosol infections were performed in a single day.

### Clinical procedures including sampling and euthanasia

Vaccines were delivered directly to the deep lung via the aerosol route in a manner similar to how the *Mtb* challenge is administered. Eight weeks post vaccination, each of the three groups was infected via the inhalation route with a target dose of 1,000 CFU of *Mtb* CDC1551. Samples were collected before vaccination, post vaccination and post infection. For the sake of clarity, results from vaccinated and infected NHPs are reported in two phases: post vaccination, which means after aerosol vaccination with either BCG or MtbΔ*sigH*, put before challenge with *Mtb* and post infection (or post challenge), that is, after challenge with *Mtb*.

A tuberculin skin test was performed before vaccination (−2 weeks), post vaccination (3 weeks) and post infection (11 weeks) as previously described by administration of mammalian tuberculin into the right eyelid^20^,^24^,^25^,^29^,^32^,^33^. Thoracic radiographs (CXRs) were acquired 2 weeks before vaccination and at 3, 7, 11 and 14 weeks post vaccination as previously described^20^,^24^,^25^,^29^,^32^,^66^. Briefly, the CXRs were scored by veterinary clinicians in a blinded manner on a subjective scale of 0–4, with a score of 0 denoting normal lung and a score of 4 denoting severe tuberculous pneumonia and pathology. Before vaccination/infection, all 21 animals received a score of 0, as their lungs were perfectly normal at this time. The following subjective scoring system was used: 0 (no pathological involvement); 1 (mild pathological involvement); 2 (moderate pathological involvement); 3 (extensive pathological involvement) and 4 (severe pathological involvement).

Blood was drawn before vaccination (−2 weeks) and then weekly thereafter for the performance of complete blood count and serum chemistry^20^,^24^,^25^,^29^,^32^. Blood collected in EDTA tubes (Sarstedt AG & co.) was used for whole-blood flow cytometry using panels described previously^67^. Blood collected in Cell Preparation tubes (Sarstedt AG & co.) was used to isolate PBMC for antigen-specific assays. BAL samples were obtained as previously described, using two washes of 40-ml sterile saline 2 weeks before vaccination and at 3, 7, 11 and 14 weeks^20^,^24^,^25^,^29^,^32^ and analysed for CFUs.

Necropsy to collect tissues was performed during euthanasia. There were two different reasons for euthanasia. In all 100% (7/7) unvaccinated control animals, as well as ∼57% (4/7) of BCG-vaccinated animals, disease progressed to an extent that humane euthanasia was deemed necessary by clinical veterinarians on this team (Fig. 8h and Supplementary Table 1). These humane end points were pre-defined in the animal-use protocol and applied as a measure of reduction of discomfort. The TNPRC Institutional Animal Care and Use Committee approved all animal-related procedures and activities. BCG-vaccinated (43% (3/7)) and MtbΔ*sigH*-vaccinated (100%) animals were either disease-free or had not progressed to an extent that required humane euthanasia till day 60. Such animals were euthanized for tissue collection via necropsy at that point (between days 60 and 62). At necropsy, lung, spleen and liver tissues were collected and processed as previously described, and CFUs were determined per gram of tissue, using four pooled lung samples per animal, each of which comprised five sections each, thus representing every lung lobe with at least one sample^20^,^24^,^25^,^29^,^32^. A subset of colonies obtained post-challenge necropsy was assayed for resistance to hygromycin to classify them as residual MtbΔ*sigH*, and subjected to PCR for *sigH* and *esat*6 to classify them as residual MtbΔ*sigH* and BCG, respectively. No evidence of residual MtbΔ*sigH* or BCG was found. End point criteria for euthanasia were previously described. Briefly, animals were euthanized if they exhibited four or more of the following: (I) a 2 °F increase in body temperature relative to pre-infection values that persisted for three or more consecutive weeks; (II) a 15% or greater loss in body weight; (III) serum CRP values greater than 10 mg ml^−1^ for two or more consecutive readings; (IV) CXR values higher than 2; (V) respiratory discomfort resulting in vocalization; (VI) significant-to-complete loss of appetite; and (VII) detectable bacilli in BAL samples. CRP values were included as criteria for euthanasia because CRP is a marker for systemic inflammation that exhibits a high degree of correlation with active TB in macaques^20^,^24^,^25^,^29^,^32^,^33^. Lung pathology at necropsy was determined as described earlier^21^,^26^,^29^,^30^,^38^ using stereological principles described by Sharpe *et al*.^27^. Briefly, lung involvement was quantified by point counting using an overlaid grid with 18.5-mm point spacing. Towards this end, digital images of three systematic random microscopic fields with an original magnification of × 2.5 per slide were employed. At least one sample from each of the four lobes of each lung was used. Intersections representing normal lung included interstitium and air space, while lesions comprised intersections with massive areas of inflammatory cells, haemorrhage, oedema, necrosis or individual to multifocal to lobar granulomas. Differences between completely normal and somewhat inflammed airspace containing localized, small zones of subacute inflammation sans fibrous or cellular encapsulation were not differentiated.

### Transcriptomics

BAL samples were obtained from animals before vaccination (−2 weeks), post vaccination (weeks 3 and 7) and post infection (weeks 11 and 14) as previously described^20^,^24^,^25^,^29^,^32^,^33^,^68^. For stabilization, 8 ml of 100% fetal bovine serum (Invitrogen, Life Sciences) was immediately added to 80 ml of the BAL sample. The sample was centrifuged at 400 r.p.m. for 10 min at 4 °C in an Allegra benchtop centrifuge. The pelleted cells were washed with cold RPMI media (Invitrogen, Life Sciences) and stored at −80 °C until analysis. For transcriptomics, total macaque RNA was isolated from total BAL obtained at the pre-vaccination and the 3 week post-vaccination time points as previously described^30^ using an RNAEasy kit (Qiagen), followed by RNA amplification (Ambion MessageAmp). The cDNA derived from the amplified RNA samples from pre-vaccination time points were labelled with Cy3, and samples from the 3-week post-vaccination time point were labelled with Cy5 (Agilent Technologies). Microarray analyses were performed as previously described by assessing the relative expression of transcripts in the Cy5 (experimental)-labelled samples relative to the Cy3(control)-labelled samples, using Agilent 4 × 44 k Rhesus Monkey microarrays^20^,^24^,^25^,^68^. Global impact of expression profiles was analysed using Database for Annotation, Visualization and Integrated Discovery (DAVID)^30^.

### iBALT

Immunofluorescence confocal microscopy was used to measure iBALT as previously described using formalin-fixed, paraffin-embedded tissue^31^,^69^.

### Flow cytometry

Flow cytometry was performed on whole-blood and BAL samples obtained from all 21 animals as previously described^25^,^33^. For T-cell phenotyping, the following antibodies were used: CD3 V500 (1:50, clone SP34-2), CD4 PerCP-Cy5.5 (1:10, clone L200), CD8 PE-TxRed (1:30, clone RPA-T8), CD28 APC (1:5, clone CD28.2), CD69 APC-Cy7 (1:20, clone FN50), CD95 PE-Cy5 (1:5, clone DX2), CD183 AL488 (1:10, clone 1C6/CXCR3), CD184 PE-Cy5 (1:5, clone 12G5), CD195 APC (1:5, clone 3A9), CD197 PE-Cy7 (1:20, clone 3D12), HLA-DR APC-Cy7 (1:75, clone L243) and Ki67 PE-Cy7 (1:50, clone B56) all purchased from BD Biosciences (San Jose, CA, USA). Flow cytometry analyses were conducted by gating first on lymphocytes followed by the elimination of B cells by gating for CD20. The remaining cells were gated for the selection of T cells using CD3, followed by gating into CD3^+^CD4^+^ and CD3^+^CD8^+^ subpopulations. The frequencies of CD4^+^ and CD8^+^ T cells expressing activation and homing markers were compared using Ki67, CXCR3, CCR5 and CCR7 (ref. 70). The levels of Foxp3^+^ were determined as a measure of the T_reg_ response. Finally, the extent of CD4^+^ and CD8^+^ cells belonging to either T_CM_ or T_EM_ relative to the naive T-cell population were measured using a combination of CD28 and CD95 markers^37^.

### Antigen-specific immune response

PBMCs isolated from whole blood collected from unvaccinated, vaccinated and infected NHPs were stimulated with *Mtb* cell wall extract (BEI Resources) for the performance of intracellular cytokine staining as previously described, using *Mtb* cell filtrate protein and cell wall extract for stimulation^57^.

### *In vitro* infection of rhesus macaque BMDMs

Rh-BMDMs were generated and infected with *Mtb* CDC1551, the isogenic MtbΔ*sigH* mutant in this strain, and BCG at an multiplicity of infection of 1:10 as previously described, for 4 h (refs 22, 46). CFUs were measured as described at different time points including 4, 24, 48, 72, 96 and 120 h. CXCL13 expression was measured using DNA microarray^25^ and quantitative RT–PCR^22^. The expression of pro-inflammatory mediators TNF-α, IL-1β and IL-6 was measured using real-time RT–PCR as described earlier^22^. The expression of specific chemokines, for example, CXCL9 and CXCL10, was measured using the Cytokine Monkey Magnetic 29-Plex Panel kit from Life-Tech, essentially as described earlier^22^.

### Statistical analyses

Statistical comparisons were performed using one-way or two-way ANOVA in GraphPad Prism with Sidak’s correction for multiple hypotheses. Analysis of transcriptome data was performed using Spotfire DecisionSite^24^,^25^,^68^ LOWESS scripts, Ingenuity Pathways Analysis and DAVID as previously described^25^. Specifically, genes with twofold or greater induction in triplicate samples from either vaccination were uploaded to DAVID, and statistically significant accumulation of terms calculated.

## Additional information

**Accession codes:** DNA microarray data have been deposited in the Gene Expression Omnibus under the Accession number GPL10183.

**How to cite this article:** Kaushal, D. *et al*. Mucosal vaccination with attenuated *Mycobacterium tuberculosis* induces strong central memory responses and protects against tuberculosis. *Nat. Commun.* 6:8533 doi: 10.1038/ncomms9533 (2015).

## Supplementary Material

Supplementary Information Supplementary Figures 1-6 and Supplementary Table 1

## References

[b1] Zumla A. . The WHO 2014 global tuberculosis report-further to go. Lancet Glob Health 3, e10–12 (2015). 25539957 10.1016/S2214-109X(14)70361-4

[b2] Kaufmann S. H. . Progress in tuberculosis vaccine development and host-directed therapies—a state of the art review. Lancet Respir. Med. 2, 301–320 (2014). 24717627 10.1016/S2213-2600(14)70033-5

[b3] Orme I. M. Prospects for new vaccines against tuberculosis. Trends Microbiol. 3, 401–404 (1995). 8564360 10.1016/s0966-842x(00)88987-8

[b4] Hingley-Wilson S. M., Sambandamurthy V. K. & Jacobs W. R. Jr. Survival perspectives from the world's most successful pathogen, *Mycobacterium tuberculosis*. Nat. Immunol. 4, 949–955 (2003). 14515128 10.1038/ni981

[b5] Kaufmann S. H. . Progress in tuberculosis vaccine development and host-directed therapies-a state of the art review. Lancet Respir. Med. 2, 301–320 (2014). 24717627 10.1016/S2213-2600(14)70033-5

[b6] Wilkie M. E. & McShane H. TB vaccine development: where are we and why is it so difficult? Thorax 70, 299–301 (2015). 25432943 10.1136/thoraxjnl-2014-205202 PMC4345977

[b7] Tameris M. D. . Safety and efficacy of MVA85A, a new tuberculosis vaccine, in infants previously vaccinated with BCG: a randomised, placebo-controlled phase 2b trial. Lancet 381, 1021–1028 (2013). 23391465 10.1016/S0140-6736(13)60177-4 PMC5424647

[b8] McShane H. . BCG: myths, realities, and the need for alternative vaccine strategies. Tuberculosis (Edinb) 92, 283–288 (2012). 22349516 10.1016/j.tube.2011.12.003 PMC5562290

[b9] Aronson N. E. . Long-term efficacy of BCG vaccine in American Indians and Alaska Natives: A 60-year follow-up study. JAMA 291, 2086–2091 (2004). 15126436 10.1001/jama.291.17.2086

[b10] Henao-Tamayo M., Ordway D. J. & Orme I. M. Memory T cell subsets in tuberculosis: what should we be targeting? Tuberculosis (Edinb) 94, 455–461 (2014). 24993316 10.1016/j.tube.2014.05.001

[b11] Garcia-Contreras L. . Immunization by a bacterial aerosol. Proc. Natl Acad. Sci. USA 105, 4656–4660 (2008). 18344320 10.1073/pnas.0800043105 PMC2290758

[b12] Barclay W. R. . Protection of monkeys against airborne tuberculosis by aerosol vaccination with bacillus Calmette-Guerin. Am. Rev. Respir. Dis. 107, 351–358 (1973). 4632221 10.1164/arrd.1973.107.3.351

[b13] Manjaly Thomas Z. R. & McShane H. Aerosol immunisation for TB: matching route of vaccination to route of infection. Trans. R Soc. Trop. Med. Hyg. 109, 175–181 (2015). 25636950 10.1093/trstmh/tru206 PMC4321022

[b14] Darrah P. A. . Aerosol vaccination with AERAS-402 elicits robust cellular immune responses in the lungs of rhesus macaques but fails to protect against high-dose *Mycobacterium tuberculosis* challenge. J. Immunol. 193, 1799–1811 (2014). 25024382 10.4049/jimmunol.1400676 PMC4119487

[b15] Larsen M. H. . Efficacy and safety of live attenuated persistent and rapidly cleared *Mycobacterium tuberculosis* vaccine candidates in non-human primates. Vaccine 27, 4709–4717 (2009). 19500524 10.1016/j.vaccine.2009.05.050 PMC3512200

[b16] Russell D. G. The evolutionary pressures that have molded *Mycobacterium tuberculosis* into an infectious adjuvant. Curr. Opin. Microbiol. 16, 78–84 (2013). 23290190 10.1016/j.mib.2012.11.007 PMC3637961

[b17] Hmama Z., Pena-Diaz S., Joseph S. & Av-Gay Y. Immunoevasion and immunosuppression of the macrophage by *Mycobacterium tuberculosis*. Immunol. Rev. 264, 220–232 (2015). 25703562 10.1111/imr.12268

[b18] Kaushal D. . Reduced immunopathology and mortality despite tissue persistence in a *Mycobacterium tuberculosis* mutant lacking alternative sigma factor, SigH. Proc. Natl Acad. Sci. USA 99, 8330–8335 (2002). 12060776 10.1073/pnas.102055799 PMC123067

[b19] Hernandez Pando R., Aguilar L. D., Smith I. & Manganelli R. Immunogenicity and protection induced by a *Mycobacterium tuberculosis* sigE mutant in a BALB/c mouse model of progressive pulmonary tuberculosis. Infect. Immun. 78, 3168–3176 (2010). 20457786 10.1128/IAI.00023-10 PMC2897378

[b20] Mehra S. . The *Mycobacterium tuberculosis* stress response factor SigH is required for bacterial burden as well as immunopathology in primate lungs. J. Infect. Dis. 205, 1203–1213 (2012). 22402035 10.1093/infdis/jis102 PMC3308902

[b21] Manganelli R. . Role of the extracytoplasmic-function sigma factor sigma(H) in *Mycobacterium tuberculosis* global gene expression. Mol. Microbiol. 45, 365–374 (2002). 12123450 10.1046/j.1365-2958.2002.03005.x

[b22] Dutta N. K. . The stress-response factor SigH modulates the interaction between *Mycobacterium tuberculosis* and host phagocytes. PLoS ONE 7, e28958 (2012). 22235255 10.1371/journal.pone.0028958 PMC3250399

[b23] Barclay W. R., Anacker R. L., Brehmer W., Leif W. & Ribi E. Aerosol-induced tuberculosis in subhuman primates and the course of the disease after intravenous BCG vaccination. Infect. Immun. 2, 574–582 (1970). 16557880 10.1128/iai.2.5.574-582.1970 PMC416053

[b24] Mehra S. . Granuloma correlates of protection against tuberculosis and mechanisms of immune modulation by *Mycobacterium tuberculosis*. J. Infect. Dis. 207, 1115–1127 (2013). 23255564 10.1093/infdis/jis778 PMC3633457

[b25] Mehra S. . The DosR regulon modulates adaptive immunity and is essential for *M. tuberculosis* persistence. Am. J. Respir. Crit. Care Med. 191, 1185–1196 (2015). 25730547 10.1164/rccm.201408-1502OC PMC4451619

[b26] Kaushal D., Mehra S., Didier P. J. & Lackner A. A. The non-human primate model of tuberculosis. J. Med. Primatol. 41, 191–201 (2012). 22429048 10.1111/j.1600-0684.2012.00536.x PMC3961469

[b27] Sharpe S. A. . Determination of lesion volume by MRI and stereology in a macaque model of tuberculosis. Tuberculosis (Edinb) 89, 405–416 (2009). 19879805 10.1016/j.tube.2009.09.002

[b28] Diedrich C. R. . Reactivation of latent tuberculosis in cynomolgus macaques infected with SIV is associated with early peripheral T cell depletion and not virus load. PLoS ONE 5, e9611 (2010). 20224771 10.1371/journal.pone.0009611 PMC2835744

[b29] Mehra S. . Reactivation of latent tuberculosis in rhesus macaques by coinfection with simian immunodeficiency virus. J. Med. Primatol. 40, 233–243 (2011). 21781131 10.1111/j.1600-0684.2011.00485.x PMC3227019

[b30] Kamath A. T. . New live mycobacterial vaccines: the Geneva consensus on essential steps towards clinical development. Vaccine 23, 3753–3761 (2005). 15893612 10.1016/j.vaccine.2005.03.001

[b31] Slight S. R. . CXCR5(+) T helper cells mediate protective immunity against tuberculosis. J. Clin. Invest. 123, 712–726 (2013). 23281399 10.1172/JCI65728 PMC3561804

[b32] Dutta N. K. . Genetic requirements for the survival of tubercle bacilli in primates. J. Infect. Dis. 201, 1743–1752 (2010). 20394526 10.1086/652497 PMC2862080

[b33] Phillips B. L. . LAG3 expression in active *Mycobacterium tuberculosis* infections. Am. J. Pathol. 185, 820–833 (2015). 25549835 10.1016/j.ajpath.2014.11.003 PMC4348466

[b34] Jayaraman P. . IL-1beta promotes antimicrobial immunity in macrophages by regulating TNFR signaling and caspase-3 activation. J. Immunol. 190, 4196–4204 (2013). 23487424 10.4049/jimmunol.1202688 PMC3622150

[b35] Manca C. . Hypervirulent *M. tuberculosis* W/Beijing strains upregulate type I IFNs and increase expression of negative regulators of the Jak-Stat pathway. J. Interferon Cytokine Res. 25, 694–701 (2005). 16318583 10.1089/jir.2005.25.694

[b36] Mayer-Barber K. D. . Host-directed therapy of tuberculosis based on interleukin-1 and type I interferon crosstalk. Nature 511, 99–103 (2014). 24990750 10.1038/nature13489 PMC4809146

[b37] Pitcher C. J. . Development and homeostasis of T cell memory in rhesus macaque. J. Immunol. 168, 29–43 (2002). 11751943 10.4049/jimmunol.168.1.29

[b38] Moyron-Quiroz J. E. . Role of inducible bronchus associated lymphoid tissue (iBALT) in respiratory immunity. Nat. Med. 10, 927–934 (2004). 15311275 10.1038/nm1091

[b39] Fairfax K. C. . IL-4-secreting secondary T follicular helper (Tfh) cells arise from memory T cells, not persisting Tfh cells, through a B cell-dependent mechanism. J. Immunol. 194, 2999–3010 (2015). 25712216 10.4049/jimmunol.1401225 PMC4495582

[b40] Phuah J. Y., Mattila J. T., Lin P. L. & Flynn J. L. Activated B cells in the granulomas of nonhuman primates infected with *Mycobacterium tuberculosis*. Am. J. Pathol. 181, 508–514 (2012). 22721647 10.1016/j.ajpath.2012.05.009 PMC3409439

[b41] Nunes-Alves C. . In search of a new paradigm for protective immunity to TB. Nat. Rev. Microbiol. 12, 289–299 (2014). 24590243 10.1038/nrmicro3230 PMC4085047

[b42] Kohlmeier J. E. . Inflammatory chemokine receptors regulate CD8(+) T cell contraction and memory generation following infection. J. Exp. Med. 208, 1621–1634 (2011). 21788409 10.1084/jem.20102110 PMC3149221

[b43] Caruso A. M. . Mice deficient in CD4 T cells have only transiently diminished levels of IFN-gamma, yet succumb to tuberculosis. J. Immunol. 162, 5407–5416 (1999). 10228018

[b44] Chen C. Y. . A critical role for CD8 T cells in a nonhuman primate model of tuberculosis. PLoS Pathog. 5, e1000392 (2009). 19381260 10.1371/journal.ppat.1000392 PMC2663842

[b45] Mehra S. & Kaushal D. Functional genomics reveals extended roles of the *Mycobacterium tuberculosis* stress response factor sigmaH. J. Bacteriol. 191, 3965–3980 (2009). 19376862 10.1128/JB.00064-09 PMC2698404

[b46] Mehra S., Dutta N. K., Mollenkopf H. J. & Kaushal D. *Mycobacterium tuberculosis* MT2816 encodes a key stress-response regulator. J. Infect. Dis. 202, 943–953 (2010). 20701538 10.1086/654820 PMC3052882

[b47] Kernodle D. S. SigH, antioxidants, and the pathogenesis of pulmonary tuberculosis. J. Infect. Dis. 205, 1186–1188 (2012). 22402036 10.1093/infdis/jis108

[b48] Winau F., Hegasy G., Kaufmann S. H. & Schaible U. E. No life without death—apoptosis as prerequisite for T cell activation. Apoptosis 10, 707–715 (2005). 16133862 10.1007/s10495-005-2940-6

[b49] Obst R. . Sustained antigen presentation can promote an immunogenic T cell response, like dendritic cell activation. Proc. Natl Acad. Sci. USA 104, 15460–15465 (2007). 17881563 10.1073/pnas.0707331104 PMC2000557

[b50] Harris J. & Keane J. How tumour necrosis factor blockers interfere with tuberculosis immunity. Clin. Exp. Immunol. 161, 1–9 (2010). 20491796 10.1111/j.1365-2249.2010.04146.x PMC2940142

[b51] Wajant H., Pfizenmaier K. & Scheurich P. Tumor necrosis factor signaling. Cell. Death Differ. 10, 45–65 (2003). 12655295 10.1038/sj.cdd.4401189

[b52] Bruns H. . Anti-TNF immunotherapy reduces CD8+ T cell-mediated antimicrobial activity against *Mycobacterium tuberculosis* in humans. J. Clin. Invest. 119, 1167–1177 (2009). 19381021 10.1172/JCI38482 PMC2673881

[b53] Harris J., Hope J. C. & Keane J. Tumor necrosis factor blockers influence macrophage responses to *Mycobacterium tuberculosis*. J. Infect. Dis. 198, 1842–1850 (2008). 18954258 10.1086/593174

[b54] Schaible U. E., Sturgill-Koszycki S., Schlesinger P. H. & Russell D. G. Cytokine activation leads to acidification and increases maturation of *Mycobacterium avium*-containing phagosomes in murine macrophages. J. Immunol. 160, 1290–1296 (1998). 9570546

[b55] Kernodle D. S. Decrease in the effectiveness of Bacille Calmette-Guerin vaccine against pulmonary tuberculosis: a consequence of increased immune suppression by microbial antioxidants, not overattenuation. Clin. Infect. Dis. 51, 177–184 (2010). 20524854 10.1086/653533

[b56] Novikov A. . *Mycobacterium tuberculosis* triggers host type I IFN signaling to regulate IL-1beta production in human macrophages. J. Immunol. 187, 2540–2547 (2011). 21784976 10.4049/jimmunol.1100926 PMC3159798

[b57] Adekambi T. . Distinct effector memory CD4+ T cell signatures in latent *Mycobacterium tuberculosis* infection, BCG vaccination and clinically resolved tuberculosis. PLoS ONE 7, e36046 (2012). 22545156 10.1371/journal.pone.0036046 PMC3335801

[b58] Riou C. . A subset of circulating blood mycobacteria-specific CD4 T cells can predict the time to *Mycobacterium tuberculosis* sputum culture conversion. PLoS ONE 9, e102178 (2014). 25048802 10.1371/journal.pone.0102178 PMC4105550

[b59] Hamilton S. E. & Jameson S. C. CD8 T cell memory: it takes all kinds. Front. Immunol. 3, 353 (2012). 23230436 10.3389/fimmu.2012.00353 PMC3515884

[b60] Lin P. L. . The multistage vaccine H56 boosts the effects of BCG to protect cynomolgus macaques against active tuberculosis and reactivation of latent *Mycobacterium tuberculosis* infection. J. Clin. Invest. 122, 303–314 (2012). 22133873 10.1172/JCI46252 PMC3248283

[b61] Ottenhoff T. H. & Kaufmann S. H. Vaccines against tuberculosis: where are we and where do we need to go? PLoS Pathog. 8, e1002607 (2012). 22589713 10.1371/journal.ppat.1002607 PMC3349743

[b62] Cruz A. . BCG vaccination-induced long-lasting control of *Mycobacterium tuberculosis* correlates with the accumulation of a novel population of CD4(+)IL-17(+)TNF(+)IL-2(+) T cells. Vaccine 33, 85–91 (2015). 25448107 10.1016/j.vaccine.2014.11.013

[b63] Hartings J. M. & Roy C. J. The automated bioaerosol exposure system: preclinical platform development and a respiratory dosimetry application with nonhuman primates. J. Pharmacol. Toxicol. Methods 49, 39–55 (2004). 14670693 10.1016/j.vascn.2003.07.001

[b64] Roy C. J. . Thermostable ricin vaccine protects rhesus macaques against aerosolized ricin: epitope-specific neutralizing antibodies correlate with protection. Proc. Natl Acad. Sci. USA 112, 3782–3787 (2015). 25775591 10.1073/pnas.1502585112 PMC4378443

[b65] Swearengen J. R. Biodefense: Research Methodology and Animal Models 2nd edn Taylor & Francis (2012).

[b66] Darrah P. A. . Aerosol vaccination with AERAS-402 elicits robust cellular immune responses in the lungs of rhesus macaques but fails to protect against high-dose *Mycobacterium tuberculosis* challenge. J. Immunol. 193, 1799–1811 (2014). 25024382 10.4049/jimmunol.1400676 PMC4119487

[b67] Dutta N. K., McLachlan J., Mehra S. & Kaushal D. Humoral and lung immune responses to *Mycobacterium tuberculosis* infection in a primate model of protection. Trials Vaccinol. 3, 47–51 (2014). 25197327 10.1016/j.trivac.2014.02.001 PMC4153710

[b68] Mehra S. . Transcriptional reprogramming in nonhuman primate (rhesus macaque) tuberculosis granulomas. PLoS ONE 5, e12266 (2010). 20824205 10.1371/journal.pone.0012266 PMC2930844

[b69] Gopal R. . S100A8/A9 proteins mediate neutrophilic inflammation and lung pathology during tuberculosis. Am. J. Respir. Crit. Care Med. 188, 1137–1146 (2013). 24047412 10.1164/rccm.201304-0803OC PMC3863739

[b70] Veazey R. S. . Dynamics of CCR5 expression by CD4(+) T cells in lymphoid tissues during simian immunodeficiency virus infection. J. Virol. 74, 11001–11007 (2000). 11069995 10.1128/jvi.74.23.11001-11007.2000 PMC113180

