# Supplementary material for: Mucosal vaccination with attenuated Mycobacterium tuberculosis induces strong central memory responses and protects against tuberculosis
Source: Nat Commun. 2015 Oct 13;6:8533. doi: 10.1038/ncomms9533 (PMC4608260; doi:10.1038/ncomms9533)
Supplement: Supplementary Information — Supplementary Figures 1-6 and Supplementary Table 1 [file ncomms9533-s1.pdf]

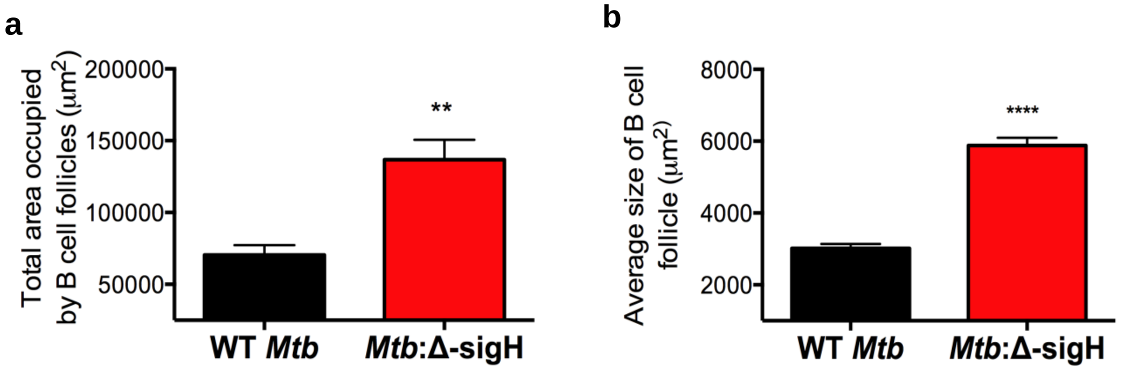

Supplemental Figure 1. iBALT formation by MtbΔ*sigH* immunization. Lung granulomas arising from infection with *Mtb* exhibit significantly reduced iBALT response relative to those from a comparable infection with MtbΔ*sigH*. (1) (**a**) Total area occupied by iBALT follicles in μm^2 in the two different groups of animals and (**b**) the average size of B cell follicles in μm^2 in the two different groups. Data from multiple lesions from six different animals was used to generate averages and statistical significance as described earlier (2), using a student's *t*-test in GraphPad Prism (**a-b**) ** $p < 0.01$, **** $p < 0.0001$.

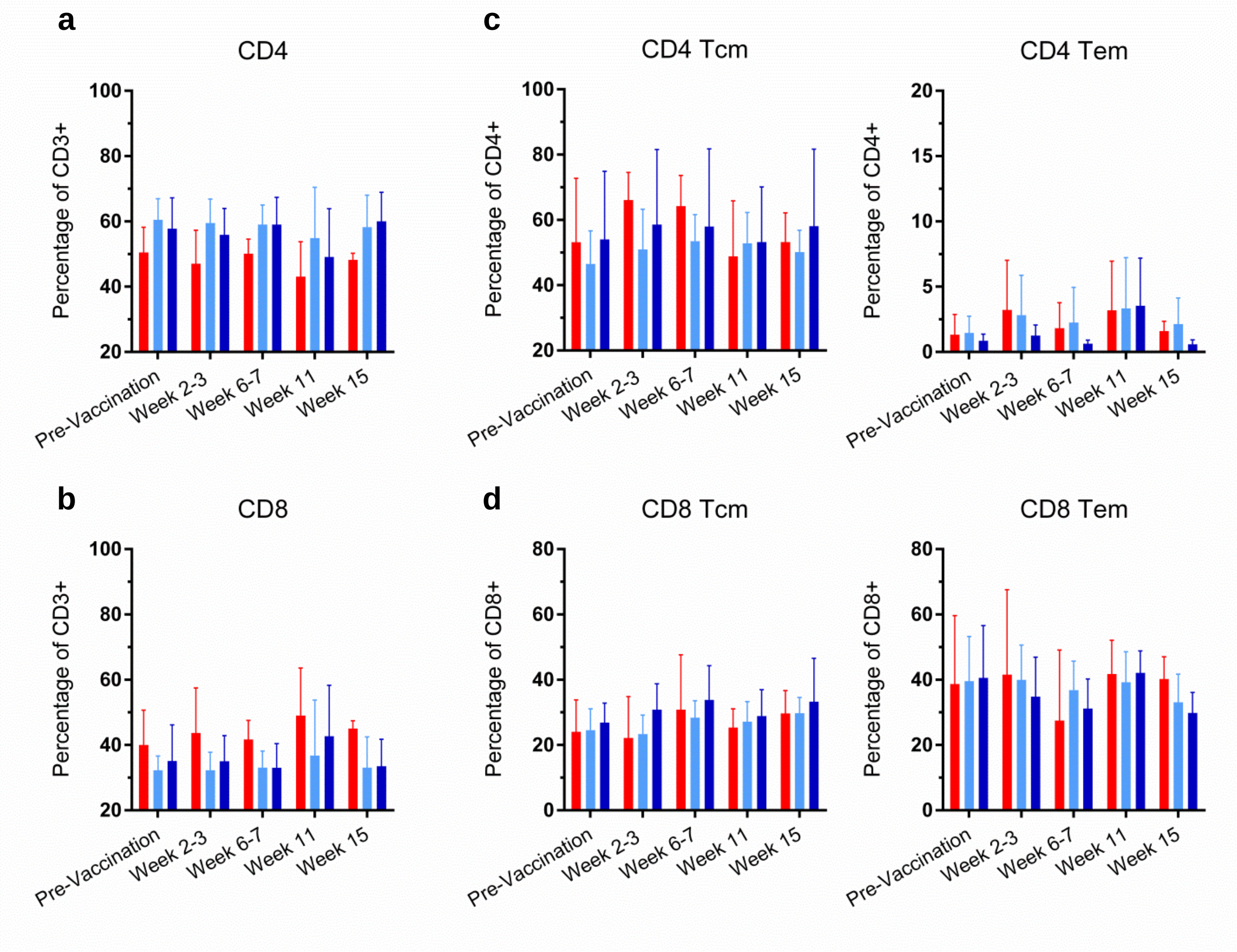

Supplementary Figure 2. Frequency of immune cells found in the periphery throughout the study. Frequency of circulating (**A**) $CD4^+$ T cells and (**B**) $CD8^+$ T cells; (**C**) $CD4^+$ T_{CM} and T_{EM} cells and of (**D**) $CD8^+$ T_{CM} and T_{EM} cells in groups of unvaccinated (**red**), BCG-vaccinated (**light blue**) and MtbΔ*sigH*-vaccinated (**dark blue**) animals. No statistical significance as determined using Two-way ANOVA. Data are means ± S.D.

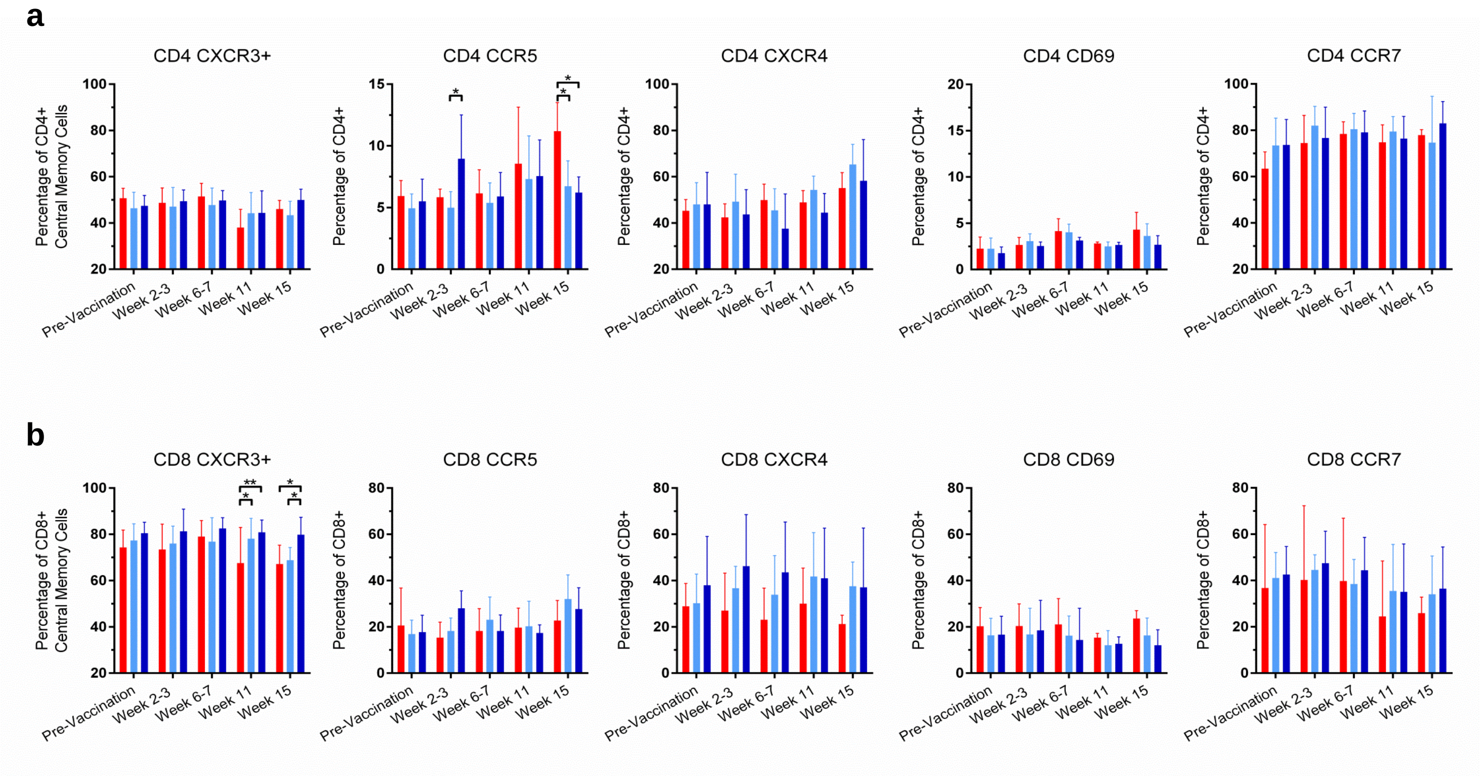

Supplementary Figure 3. Functional phenotype of immune cells found in the periphery throughout the study. Frequency of functional phenotype, determined by chemokine receptors CXCR3, CCR5, CXCR4, and CCR7 and activation maker CD69, circulating (**A**) $CD4^+$ T cells and (**B**) $CD8^+$ T cells in groups of unvaccinated (**red**), BCG-vaccinated (**light blue**) and MtbΔ*sigH*-vaccinated (**dark blue**) animals. *P < 0.05, **P < 0.01, ***P < 0.001, ****P < 0.0001 using two-way ANOVA. Data are means ± S.D

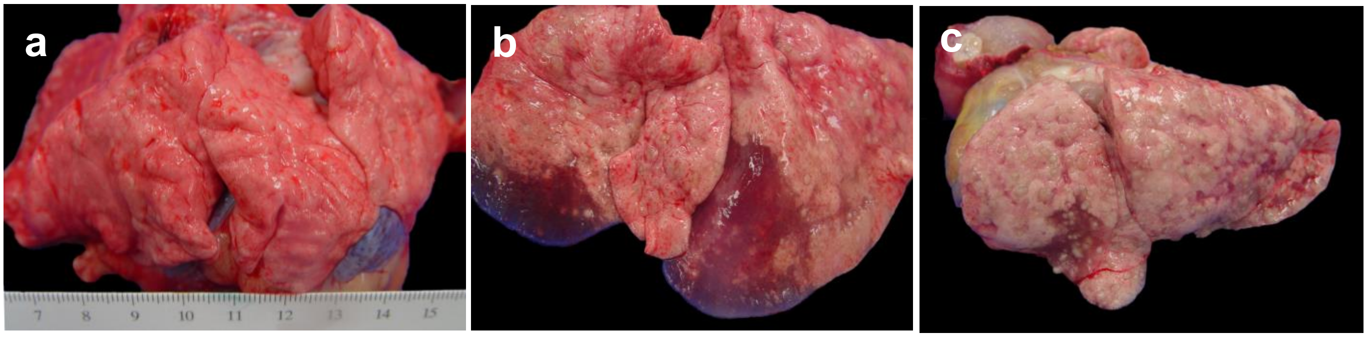

Supplementary Figure 4. Gross and histopathology of lung samples from representative animals of the three groups. MtbΔ*sigH* vaccinated (**a**), BCG-vaccinated (**b**) and Unvaccinated (**c**). Gross images of lung were edited by altering the background to solid black, cropping out the animal label as well as most or all of the empty space around the tissue, and then enlarging by 2x.

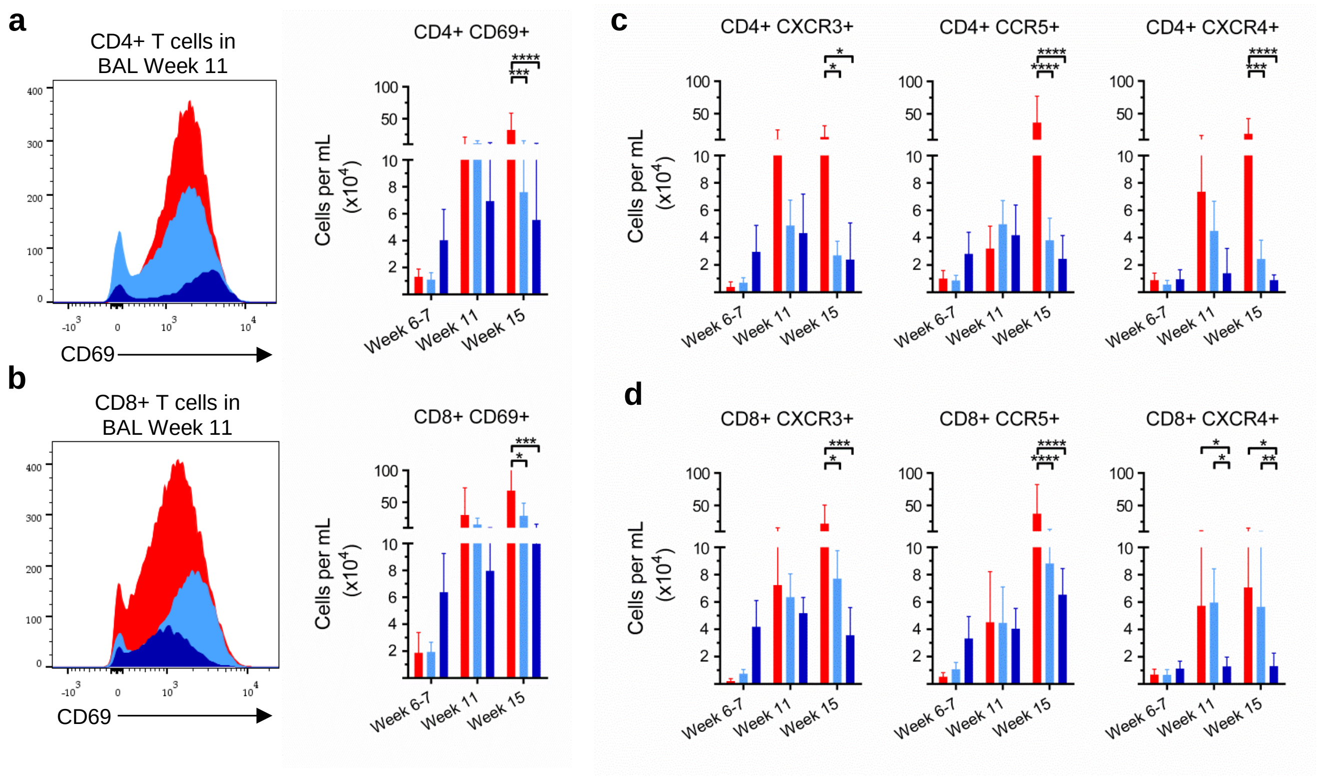

Supplementary Figure 5.

(**a**,**b**) Representative histogram of absolute cell count at week 15 of CD4$^+$CD69$^+$ (**a**) and CD8$^+$CD69$^+$ (**b**) T cells and their numbers after challenge in BAL. (**c**,**d**) Absolute count of cells per mL of BAL at different stages of infection of phenotypic markers CXCR3, CCR5, and CXCR4 in CD4$^+$ (**c**) and CD8$^+$ (**d**) T cells. *P < 0.05 **P < 0.01, ***P < 0.001, ****P < 0.0001 using two-way ANOVA. Data are means ± S.D. Unvaccinated (**red**), BCG-vaccinated (**light blue**) and MtbΔ*sigH*-vaccinated (**dark blue**).

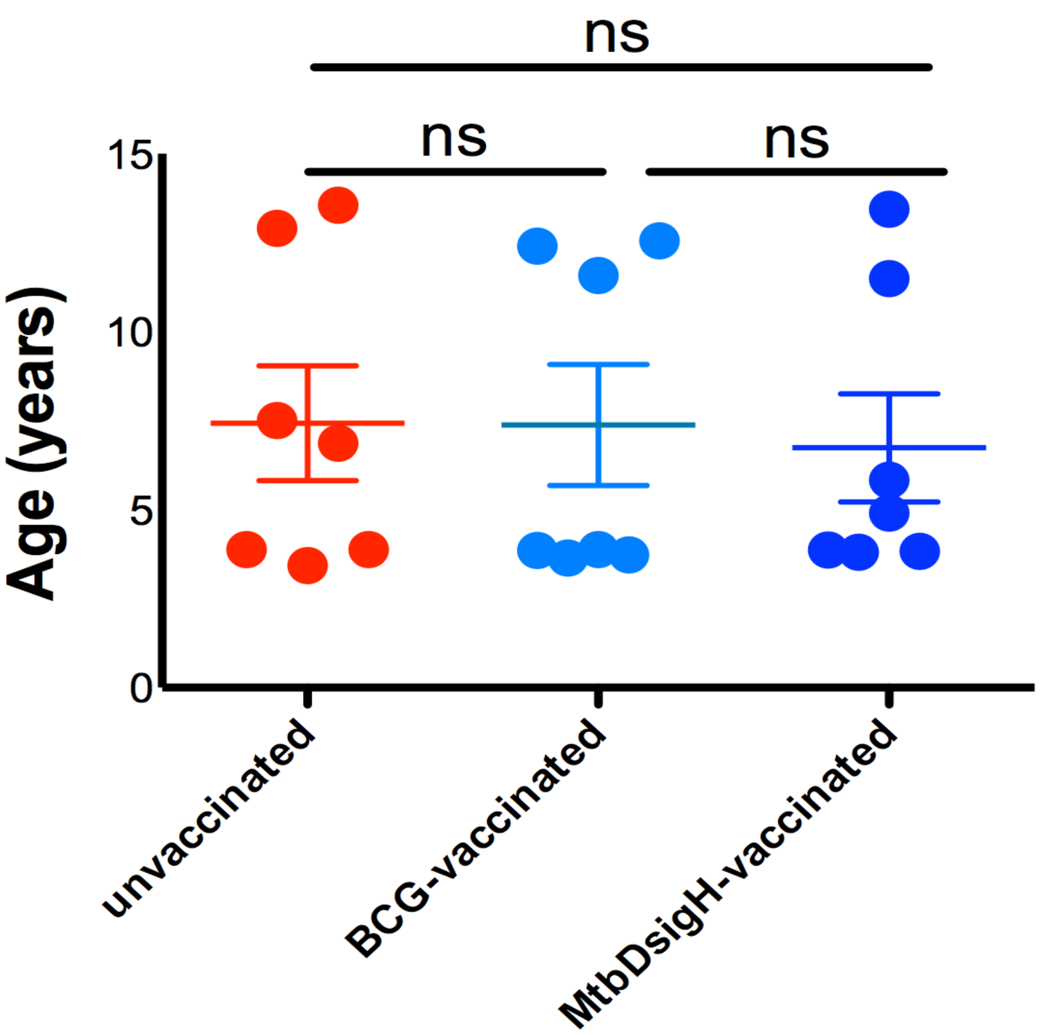

Supplementary Figure 6.

Age in years of macaques on the study and their representative distribution into the three different subject groups: unvaccinated (**red**), BCG-vaccinated (**light blue**) and MtbΔ*sigH*-vaccinated (**dark blue**). There was no statistically significant different in the average age of the animals in any of the groups, which were 7.42 years (unvaccinated), 7.38 years (BCG-vaccinated) and 6.75 years (MtbΔ*sigH*-vaccinated).

Supplemental Table 1.

Animal ID	Vaccination group	Time-to-humane-euthanasia (days from vaccination)	TST (pre-vaccination)	TST (post-vaccination)	TST (post-challenge)
GK87	Unvaccinated	38	NNN	NNN	PPP
CL10	Unvaccinated	51	NNN	NNN	PPP
DF30	Unvaccinated	34	NNN	NNN	PPP
II46	Unvaccinated	31	NNN	NNN	PPP
II43	Unvaccinated	59	NNN	NNN	PPP
IL11	Unvaccinated	42	NNN	NNN	PPP
GI89	Unvaccinated	59	NNN	NNN	PPP
CR57	BCG	51	NNN	PPP	PPP
EA97	BCG	NA	NNN	PPP	PPP
IL33	BCG	39	NNN	PPP	PPP
IJ42	BCG	44	NNN	PPP	PPP
II21	BCG	46	NNN	PPP	PPP
HE06	BCG	NA	NNN	PPP	PPP
HN69	BCG	NA	NNN	PPP	PPP
FM29	MtbΔ*sigH*	NA	NNN	PPP	PPP
DF44	MtbΔ*sigH*	NA	NNN	PPP	PPP
CK53	MtbΔ*sigH*	NA	NNN	PPP	PPP
IJ23	MtbΔ*sigH*	NA	NNN	PPP	PPP
IM73	MtbΔ*sigH*	NA	NNN	PPP	PPP
IN85	MtbΔ*sigH*	NA	NNN	PPP	PPP
II07	MtbΔ*sigH*	NA	NNN	PPP	PPP

Footnotes:

1. All animals were infected with the *Mtb* CDC1551 strain via aerosol.
2. N and P indicate if the test was negative or positive. Macaques were assessed for positivity to tuberculin 24, 48 and 72 hrs after each administration. Hence NNN indicates that the animal was negative for TST at all three time points while PPP indicates a positive response was elicited at each of the three times points.
3. NA – not applicable; macaque survived till day 60, the duration of experimental protocol and was experimentally euthanized for collection of time-compared tissues.
